# Targeted next generation sequencing can serve as an alternative to conventional tests in myeloid neoplasms

**DOI:** 10.1371/journal.pone.0212228

**Published:** 2019-03-06

**Authors:** Borahm Kim, Hyeonah Lee, Jieun Jang, Soo-Jeong Kim, Seung-Tae Lee, June-Won Cheong, Chuhl Joo Lyu, Yoo Hong Min, Jong Rak Choi

**Affiliations:** 1 Department of Laboratory Medicine, Yonsei University College of Medicine, Seoul, Korea; 2 Brain Korea 21 PLUS Project for Medical Science, Yonsei University, Seoul, Korea; 3 Department of Internal Medicine, Yonsei University College of Medicine, Seoul, Korea; 4 Department of Pediatrics, Yonsei University College of Medicine, Seoul, Korea; Ohio State University Wexner Medical Center, UNITED STATES

## Abstract

The 2016 World Health Organization classification introduced a number of genes with somatic mutations and a category for germline predisposition syndromes in myeloid neoplasms. We have designed a comprehensive next-generation sequencing assay to detect somatic mutations, translocations, and germline mutations in a single assay and have evaluated its clinical utility in patients with myeloid neoplasms. Extensive and specified bioinformatics analyses were undertaken to detect single nucleotide variations, *FLT3* internal tandem duplication, genic copy number variations, and chromosomal copy number variations. This enabled us to maximize the clinical utility of the assay, and we concluded that, as a single assay, it can be a good supplement for many conventional tests, including Sanger sequencing, RT-PCR, and cytogenetics. Of note, we found that 8.4–11.6% of patients with acute myeloid leukemia and 12.9% of patients with myeloproliferative neoplasms had germline mutations, and most were heterozygous carriers for autosomal recessive marrow failure syndromes. These patients often did not respond to standard chemotherapy, suggesting that germline predisposition may have distinct and significant clinical implications.

## Introduction

Whole genome and exome studies have revealed a wide genetic heterogeneity in myeloid neoplasm by discovering oncogenic mutations in hundreds of genes [1]. Embracing this advancement in knowledge, revised 2016 World Health Organization (WHO) classification has incorporated or suggested a number of genes required for the diagnosis and risk stratification of myeloid neoplasms [2]. Of note, the WHO 2016 classification has added a section on myeloid neoplasms with a germline predisposition for use in classifying cases with an inherited defect in genes for platelet disorder, bone marrow failure syndrome (MFS), Noonan syndrome, telomere biology disorder, and many others: there are more than a hundred genes responsible for this category.

Many clinical laboratories are adopting next-generation sequencing (NGS) panel testing in diagnosis of patients with acute leukemia. Using NGS testing, a number of mutations that are critical in diagnosis and risk stratification can be identified in a relatively short time, compared to conventional tests, although conventional testing methods, such as fluorescence in situ hybridization (FISH) and RT-PCR, still have their strengths in detecting various type of genetic variations, including structural variations. As application of NGS testing has broadened discoveries of unexpected genetic variations, comprehensive understanding of disease is becoming possible, with which better prognostication and treatment choices are expected.

With the increasing demand for an ability to examine a variety of genes, we have designed a comprehensive genetic test using NGS for acute myeloid leukemia (AML) and other myeloid neoplasms, reflecting changes in the new WHO 2016 classification, that covers genes for most germline predisposition syndromes and intronic hotpots of 12 recurrently translocated genes. To maximize the utility of the assay, we conducted extensive bioinformatics analyses: one useful approach was to recycle off-target data to analyze whole-genome copy number status, which could supplement conventional cytogenetics. We have deemed that our comprehensive test, as a single assay, could be a good substitute or supplement for many conventional tests.

Utilizing our newly developed test, we were able to discover a high frequency of germline mutations in cancer predisposition genes. Patients with these mutations exhibited different clinical characteristics suggesting that germline predisposition has distinct and significant clinical implications.

## Materials and methods

### Patients and samples

Bone marrow aspirates of patients diagnosed with AML, myelodysplastic syndrome (MDS), and myeloproliferative neoplasm (MPN) in our center between July 2016 and May 2017 were obtained after getting informed consent for genetic study from each patient. The current study was approved by Severance Hospital Institutional Review Board (4-2016-0869).

### Conventional laboratory tests

Conventional G-banding karyotyping and FISH were performed using heparinized bone marrow aspirate following standard protocols. FISH for *BCR-ABL1*, *PML-RARA*, *RUNX1-RUNX1T1*, *KMT2A*, and *CBFB-MYH11* were performed using Vysis probes (Abbott Molecular, Abbott Park, IL, USA). To identify recurrent translocations, RT-PCR was performed using a HemaVision kit (DNA Technology, Aarhus, Denmark) according to the manufacturer’s instructions. For *FLT3* internal tandem duplication (ITD) detection, PCR amplification and fragment analysis were conducted using a 3130 DNA Analyzer (Applied Biosystems, Foster City, CA, USA) and Gene-Mapper 3.2 software (Applied Biosystems). Sanger sequencing was performed using the BigDye Terminator Cycle Sequencing Ready Reaction Kit on an ABI Prism 3730 Genetic Analyzer (Applied Biosystems).

### Gene panel and probe design

After reviewing the WHO 2016 classification and other related literature, a total of 215 genes were included in the panel (S1 Table). They include 116 genes frequently mutated or rearranged in myeloid neoplasm and 113 genes in which variations are known to predispose myeloid neoplasm, with 14 genes overlapping. All coding exons were included. Intronic regions with reported pathogenic mutations were retrieved from the ClinVar database (version 20170502) and HGMD professional (version 16.02) and were added to the target regions. To detect gene rearrangements at the DNA level, we included intronic breakpoint hotspots for 12 recurrently translocated genes to the target regions: *BCR*, *FGFR1*, *FUS*, *JAK2*, *KMT2A*, *MYH11*, *NUP214*, *PDGFRA*, *PDGFRB*, *RARA*, *RBM15*, and *RUNX1* (S2 Table).

Complementary RNA probes, approximately 120 bp in length, were designed to 2× tile across target genes and were synthesized (Celemics, Seoul, Korea). Probes for 18 core genes and regions with high mutation frequency and/or clinical implications, such as *NPM1* exon 11 and *FLT3* exons 14 and 15, were designed to have an ultra-high sequencing depth (S3 Table). Repeat masking was not performed to avoid the possibility of missing translocations that occurred in repeat areas. In total, the size of the capture region was estimated to be 0.8 Mb.

### Capture and sequencing

Genomic DNA was extracted from bone marrow using a QIAamp DNA Blood Mini Kit (Qiagen, Venlo, The Netherlands). Approximately 1.5 μg of genomic DNA was fragmented to segments between 150 and 250 bp in length using the Bioruptor Pico Sonication System (Diagenode, Belgium). The resulting DNA was then end-repaired and ligated to Illumina adapters (Illumina, San Diego, CA, USA). Sequence indexes were added to the samples to allow all samples to be sequenced in a single flow cell. Small fragments of ~100 bp and unligated adapters were removed using the AMPure purification system (Agencourt Bioscience, Beverly, MA, USA). Sequencing libraries were then hybridized with the capture probes. Streptavidin-coated paramagnetic beads were used to remove unbound DNA. The captured DNA was finally eluted from the magnetic beads by digestion of the cRNA capture probes and purified. The enriched DNA was then amplified using universal primers targeting the paired-end adapters, clusters were generated, and DNA was sequenced on a NextSeq 550 instrument (Illumina) with 2×151 bp reads. All procedures were performed according to the manufacturer’s instructions.

### Data analysis

**Fig 1:**
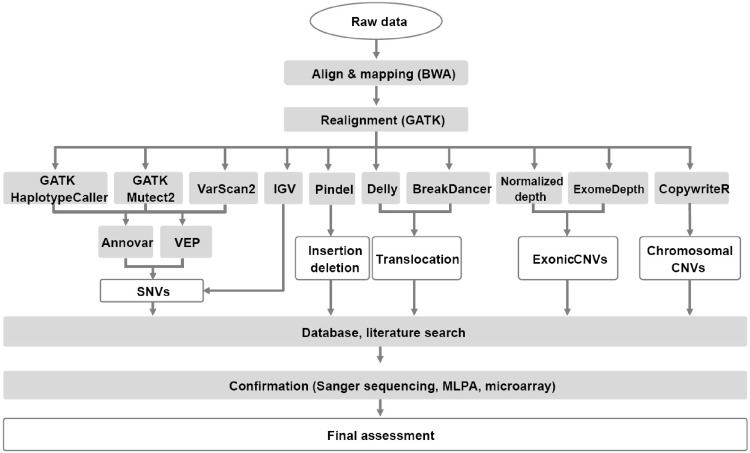
NGS data analysis flow and bioinformatics tools.

The analysis flow and bioinformatic tools used are depicted in Fig 1. Reads were aligned to human genomic reference sequences (GRCh37) using the Burrows-Wheeler alignment (BWA) tool (version 0.7.12)[3]. To identify single nucleotide variations (SNV) and insertion and deletions (indels), HaplotypeCaller and MuTect2 in the GATK package (3.8–0) and VarScan2 (2.4.0) were used, and the results of the three algorithms were compared and merged [4–6]. A split-read analysis was conducted using Pindel (version 0.2.0) to detect large indels, especially *FLT3* ITDs [7]. Translocations were identified using BreakDancer (1.3.6) and Delly2 (version 0.7.7) [8,9]. All mutations were annotated using ANNOVAR and VEP (87) software [10,11]. Annotated variants were further evaluated using the following filtering strategy: 1) variants classified as benign and likely benign according to the Standards and Guidelines by the American College of Medical Genetics and Genomics (ACMG) and the Association for Molecular Pathology (AMP) [12], with a scoring algorithm implemented in the DxSeq Analyzer (Dxome, Seoul, Korea), were excluded; 2) variants with > 0.01 population frequency judged using the Exome Aggregation Consortium (ExAC) database were eliminated; 3) variants present as a somatic mutation in the COSMIC database of cancer mutations were included; and 4) known mutations of recurrently mutated genes in myeloid neoplasms were included. Most missense variants with unknown significance were discarded, and nonsense, frameshift, or splice site variants were included when the known mechanism of the mutation was loss-of-function. All of the variants were manually verified using the Integrative Genomic Viewer [13].

An R package, ExomeDepth (version 1.1.10), was used to detect genic or exonic level copy number variations (CNV) in target regions, followed by visualization using a base-level read depth normalization algorithm implemented in the DxSeq Analyzer (Dxome)[14]. The R package CopywriteR (version 2.9.0) was used with a 1 Mb window option for off-target analysis and whole chromosomal CNV detection [15].

### Confirmation with other methods

For 34 cases of AML and 16 cases of MPN, Sanger sequencing was additionally performed for 11 genes with a high mutation frequency: these included *ASXL1*, *CEBPA*, *CALR*, *DNMT3A*, *FLT3*, *IDH1*, *IDH2*, *JAK2*, *KIT*, *MPL*, and *NPM1*. Highly probable CNVs noted upon visual inspection were further confirmed by multiplex ligation-dependent probe amplification (MLPA) if probes were available from MRC-Holland (Amsterdam, The Netherlands). Pathogenic mutations in germline predisposition genes were confirmed by the Sanger sequencing of buccal swab or peripheral blood samples at complete remission.

## Results

### Patients and diagnosis

A total of 129 patients comprising 57 females and 72 males (median age of 56 years; ranging from 6 months to 85 years) were enrolled. Ninety-five were diagnosed with AML, 31 were diagnosed with MPN, and three were diagnosed with MDS (Table 1).

**Table 1:** WHO classification of cases enrolled in this study.

WHO classification	n
**Myeloproliferative neoplasms (MPN)**	
Polycythemia vera	5
Primary myelofibrosis	8
Essential thrombocythemia	18
**Myelodysplastic syndrome (MDS)**	
Refractory anemia with excess blasts	3
**Acute myeloid leukemia (AML) with recurrent genetic abnormalities**	
AML with t(8;21)(q22;q22); *RUNX1*-*RUNX1T1*	9
AML with inv(16)(p13.1q22) or t(16;16)(p13.1;q22); *CBFB*-*MYH11*	3
APL with t(15;17)(q22;q12); *PML*-*RARA*	9
AML with t(9;11)(p22;q23); *MLLT3*-*KMT2A*	1
AML with mutated *NPM1*	14
AML with biallelic mutations of *CEBPA*	8
**AML with myelodysplasia-related changes**	14
**Therapy-related myeloid neoplasms**	1
**AML, NOS**	
AML without maturation	3
AML with maturation	27
Acute myelomonocytic leukemia	3
Acute monoblastic/monocytic leukemia	2
**Myeloid proliferations related to Down syndrome**	1
**Total**	**129**

### NGS statistics

On average, 14.36 ± 3.05 million reads were generated per sample, with approximately 98% (13.97 ± 3.31 million reads) being mapped to the human reference genome (GRCh37). Mean coverage within target regions was 670×, and for every sample, more than 99.7% of the target region was covered with at least 30 reads.

### Sequence variations

**Fig 2:**
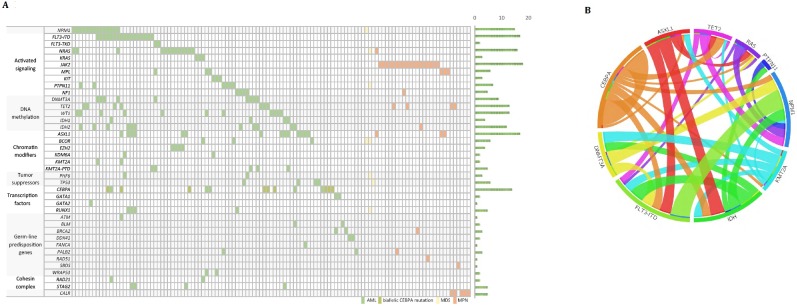
Characterization of mutations. (A) Mutations classified according to the categories of gene functions. (B) Circos diagram showing co-occurrence and mutual exclusivity of mutations.

After filtering out benign or likely benign polymorphisms, we identified 280 variants involving 65 genes (Fig 2 and S4 Table). At least one variant was detected in 119 patients, and on average, 2.4 variants were detected per patient. For 34 AML and 16 MPN patients, 11 frequently mutated genes were sequenced by NGS and Sanger sequencing all in perfect agreement (S5 Table).

We observed a relatively high frequency of mutations in genes involved in signal transduction and epigenetic regulation. In addition to *FLT3* and *NPM1* mutations, we found a high frequency of *ASXL1* mutations in AML cases, somewhat more frequent than what has been reported previously [1,16–18]. Among 14 patients with *CEBPA* mutation, biallelic mutations were found in nine patients. In MPN patients, *JAK2* V617F mutation was most common, followed by mutations in *ASXL1*, *MPL*, and *TET2*. We found a concurrence among *DNMT3A* and *NPM1* mutations and *FLT3* ITD as previously reported (Fig 2) [19]. Mutations in epigenetic modifiers, including *DNMT3A*, *ASXL1*, *IDH1/2*, and *TET2*, tended to occur with other mutations, in line with the hypothesis that mutations in these genes are early events [20–24]. *NPM1* mutations were often accompanied with mutations in these epigenetic genes [24] and were associated with mutations in *NRAS* codon 12 and codon 13.[24] Mutations in genes of the same pathway rarely coexisted. *WT1* mutations were more common in patients younger than 60 years and were less likely to coexist with *DNMT3A*, *ASXL1*, *IDH1*, and *IDH2* mutations. Also, multiple subclones were commonly observed, in line with previous observations [16,17,25].

### *FLT3* ITD

By conventional PCR and fragment analysis, 17 cases were found to have *FLT3* ITD mutation. GATK, an algorithm optimized for the detection of SNVs, could detect the mutation in only 8 (47%) of 17 positive cases. Pindel, an algorithm based on split-read identification and optimized for indel detection, could detect mutations in all cases. Table 2 summarizes *FLT3* ITD results with duplication size and allele frequency. Insertion sites were exclusively located in exon 14, a juxtamembrane domain, with inserted sequences of varying sizes (24–201 bp) and allele frequency (2.3–30.3%).

**Table 2:** Identification of *FLT3*- ITD by different NGS algorithms.

ID	PCR-fragment analysis	NGS, GATK	NGS, Pindel		
			Mutation	Duplication size (bp)	Allele frequency
P1	Detected	-	Detected	183	0.07
P5	Detected	-	Detected	39	0.22
P6	Detected	-	Detected	120	0.02
P7	Detected	Detected	Detected	42	0.12
P8	Detected	-	Detected	84	0.04
P9	Detected	Detected	Detected	66	0.12
P10	Detected	-	Detected	201	0.18
P14	Detected	Detected	Detected	54	0.34
P41	Detected	-	Detected	87	0.19
P77	Detected	-	Detected	24	0.15
P84	Detected	-	Detected	96	0.03
P91	Detected	Detected	Detected	57	0.15
P93	Detected	Detected	Detected	48	0.10
P95	Detected	-	Detected	138	0.10
P115	Detected	Detected	Detected	54	0.30
P128	Detected	Detected	Detected	43	0.17
P130	Detected	Detected	Detected	69	0.26

#### Genic CNV

**Fig 3:**
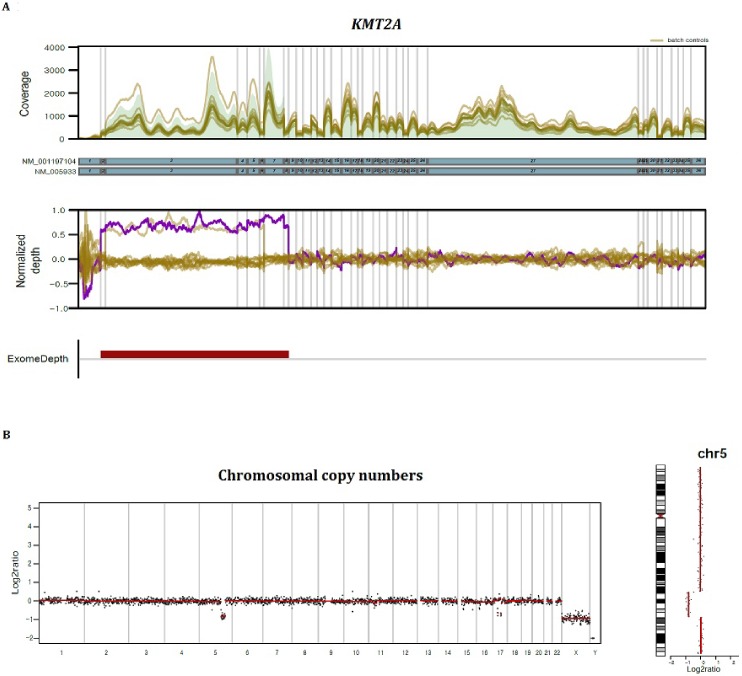
Visualization of copy number analyses. (A) CNV analysis at the genic or exonic level was performed by comparison of read depths at base level. An example of a *KMT2A* (*MLL*)-partial tandem duplication is illustrated. (B) CNV at the whole genome level was estimated by off-target read analysis. Cryptic deletion on 7q was identified, and the case could be reclassified as AML with myelodysplasia-related changes after NGS analysis.

CNV analysis at the genic or exonic level was performed by comparison of read depths in each sample to those in other samples in the same batch. Calling of abnormal copy numbers were done using R package Exomedepth [14], while illustration of depth in comparison with other samples was done using DxSeq analyzer (Dxome). Fig 3A shows an example of a *KMT2A* (*MLL*) partial tandem duplication (PTD). The deletion or duplication of exons or whole genes were detected in 19 patients, and in those genes with an available MLPA kit, the CNVs were confirmed to be true (Table 3). The results were found to be reliable, especially when changes appeared in consecutive exons, although deletion or duplication of a single exon was also confirmed to be true positive (e.g., *PALB2* exon 8 deletion in P13).

**Table 3:** Genic and exonic CNVs identified.

ID	Gene	Region	Deletion/duplication	MLPA
P4	*KMT2A*	Exons 3–6	PTD	Confirmed
P17	*KMT2A*	Exons 2–8	PTD	Confirmed
P78	*KMT2A*	Exons 2–8	PTD	Confirmed
P130	*KMT2A*	Exons 3–6	PTD	ND
P133	*KMT2A*	Exons 2–8	PTD	ND
P13	*PALB2*	Exon 8	Deletion	Confirmed
P9	*ABL1*	Whole gene	Deletion	ND
P16	*WT1*	Exons 1–9	Deletion	Confirmed
	*MYC*	Whole gene	Deletion	ND
P12	*PML*	Exons 6–8	Deletion	ND
P22	*NF1*	Whole gene	Deletion	Confirmed
P26	*EZH2*	Whole gene	Deletion	Confirmed
	*KMT2C*	Whole gene	Deletion	ND
P34	*CSF2RA*	Whole gene	Deletion	Confirmed
P36	*CBL*	Exons 8–9	Deletion	ND
P42	*RUNX1*	Exons 2–7	Duplication	ND
P47	*CSF2RA*	Whole gene	Deletion	Confirmed
P52	*NF1*	Whole gene	Duplication	Confirmed
P57	*RIT1*	Exons 1–3	Duplication	ND
P60	*TET2*	Whole gene	Deletion	ND
P118	*TP53*	Whole gene	Deletion	ND
	*SUZ12*	Whole gene	Deletion	ND

PTD, partial tandem duplication; MLPA, Multiplex ligation-dependent probe amplification; ND, not done

### Chromosomal CNV

Genome wide copy number status was assessed using R package CopywriteR [15]. Because the on-target capture efficiency was about 40% on average, CNV at the whole genome level could be estimated with the remaining off-target reads. After excluding 25 cases with incomplete cytogenetic information, such as no mitotic cells, complex karyotypes, and balanced translocations, which intrinsically cannot be detected by NGS analysis, we estimated the concordance rate between cytogenetics and NGS to be about 87.5% (91/104) (S6 Table). With higher resolution than conventional karyotyping, NGS analysis could detect small interstitial deletions, except for abnormalities in minor clones. Fig 3 shows exemplary cases that had a cryptic deletion in 5q or 7q and could be reclassified as AML with myelodysplasia-related changes after the NGS analysis. Chromosome analysis could not be completed in some cases because no mitotic cells were obtainable; therefore, NGS analysis could help in acquiring karyotype information.

### Detection of translocations at the DNA level

**Fig 4:**
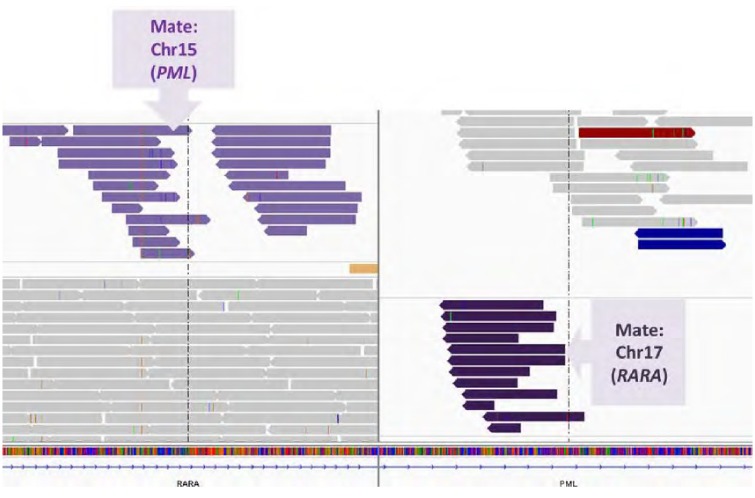
With capture probes targeting intronic breakpoints, DNA sequencing could detect recurrent translocations. An example of a case with *PML*-*RARA* fusion. The other part (mate) of each paired-end read is located in different genes in different chromosomes.

Gene rearrangements were evaluated in sequencing reads of intronic hotspots using BreakDancer and the Delly algorithm. In 26 patients with AML, the recurrent translocations *BCR-ABL1*, *RUNX1*-*RUNX1T1*, *CBFB-MYH11*, *PML-RARA*, *SET-NUP24*, *ZBTB16-RARA*, *KMT2A-MLLT3*, and *FUS-ERG* were identified by RT-PCR. Among those gene fusions, 3 (11.5%) and 17 (65.4%) cases were identified by BreakDancer and Delly, respectively (Table 4 and Fig 4).

**Table 4:** Detection of recurrent translocations in DNA samples by different NGS algorithms.

ID	Chromosome	RT-PCR	NGS, BreakDancer	NGS, Delly
P9	46,XY[20]	*SET*-*NUP214*	Detected	Detected
P11	45,X,-Y,t(8;21)(q22;q22)[20]/46,XY[4]	*RUNX1*-*RUNX1T1*	-	Detected
P12	46,XY,t(15;17)(q24;q21)[7]	*PML*-*RARA*	-	Detected
P13	46,XX,t(15;17)(q24;q21)[19]/46,XX[1]	*PML*-*RARA*	-	-
P14	47,XX,+8,t(15;17)(q24;q21)[20]	*PML*-*RARA*	-	Detected
P15	46,XY,t(11;17)(q23;q21)[3]/47,sl,+8[7]/46,XY[10]	*ZBTB16*-*RARA*	-	-
p18	46,XX[20]	*KMT2A*-*MLLT3*	-	-
P19	Not tested	*CBFB*-*MYH11*	-	-
P20	46,XX,+del(1)(p13),-16,der(21)t(16;21)(p11;q22)	*FUS*-*ERG*	-	Detected
P23	45,X,-Y,t(8;21)(q22;q22)[20]	*RUNX1*-*RUNX1T1*	-	Detected
P26	47,XX,+8,inv(16)(p13q22)[22]	*CBFB*-*MYH11*	Detected	Detected
P27	No mitotic cells	*RUNX1*-*RUNX1T1*	-	Detected
P34	46,XY[20]	*RUNX1*-*RUNX1T1*	-	Detected
P36	46,XX,inv(16)(p13q22)[19]/46,XX[9]	*CBFB*-*MYH11*	Detected	Detected
P43	46,XY,del(9)(q22)[6]/46,XY[17]	*RUNX1*-*RUNX1T1*	-	Detected
P45	46,XX,t(15;17)(q24;q21)[22]/46,XX[2]	*PML*-*RARA*	-	Detected
P47	45,X,-Y,t(8;21)(q22;q22)[21]	*RUNX1*-*RUNX1T1*	-	Detected
P72	46,XX,t(8;21)(q22;q22)[20]	*RUNX1*-*RUNX1T1*	-	-
P73	46,XX,t(8;21)(q22;q22)[20]	*RUNX1*-*RUNX1T1*	-	Detected
P82	46,XY,t(15;17)(q24;q21)[5]/46,idem,del(7)(q22)[7]/46,XY[1]	*PML*-*RARA*	-	Detected
P83	46,XY,t(15;17)(q24;q21)[16]/46,XY[4]	*PML*-*RARA*	-	Detected
P84	46,XY,t(15;17)(q24;q21)[16]/46,XY[4]	*PML*-*RARA*	-	Detected
P117	46,XX[20]	*PML*-*RARA*	-	-
P123	46,XY,t(8;21)(q23;q22)[11]/46,XY[9]	*RUNX1-ZFPM2*	-	-
P131	46,XX,t(15;17)(q24;q21)[20]	*PML*-*RARA*	-	-
P134	47,XX,+8,t(10;11)(p13;q21)[20]	*KMT2A-MLLT10*	-	-

### Germline predisposition

After examining 113 cancer predisposition genes, we could identify definite or highly probable germline mutations in nine genes, including *ATM*, *BLM*, *BRCA2*, *DDX41*, *FANCA*, *PALB2*, *RAD51*, *SBDS*, and *WRAP53*. Among 95 patients with AML, eight were confirmed to have pathogenic mutations in germline samples and three had pathogenic mutations in genes where almost only germline mutations are implicated in AML (Table 5). In total, we determined that 8.4–11.6% of patients with AML harbored germline mutations. Most patients were monoallelic mutation carriers for autosomal recessive MFSs, including Fanconi anemia, dyskeratosis congenita, Bloom syndrome, and ataxia-telangiectasia. Two patients harbored heterozygous mutations in *DDX41*, which is known as one of the frequently mutated germline predisposition genes in late onset MDS or AML. Two patients shared the same mutation, A500Cfs*9, which is reported only in Asians [26,27]. Among nine patients whose bone marrow studies were performed after induction chemotherapy, only five of them reached complete remission, suggesting a possible association between those mutations and a poor treatment response.

**Table 5:** Germline mutations identified in patients with AML and MPN.

ID	Disease	Age/Sex	Gene^†^	cDNA	Amino acid	Allele frequency	Associated disease	Inheritance pattern	BM, day 28
P5*	AML	62/M	*ATM*	c.5288_5289insGA	p.Tyr1763*	0.52	Ataxia-telangiectasia	AR	CR
P81*	AML	3/M	*BRCA2*	c.8912delA	p.Lys2971Serfs*5	0.5	Fanconi anemia, hereditary breast and ovarian cancer	AR, AD	ND
P75*	AML	67/M	*DDX41*	c.1496dupC	p.Ala500Cysfs*9	0.47	Myeloproliferative/lymphoproliferative neoplasm	AD	PR
P99*	AML	62/M	*DDX41*	c.1496dupC	p.Ala500Cysfs*9	0.46	Myeloproliferative/lymphoproliferative neoplasm	AD	CR
P30*	AML	12/M	*FANCA*	c.1A>T	p.Met1?	0.46	Fanconi anemia	AR	PR
P35*	AML	26/F	*PALB2*	c.1011_1015delACCAG	p.Leu337Phefs*3	0.44	Fanconi anemia, hereditary breast and ovarian cancer	AR, AD	PR
P34*	AML	17/M	*WRAP53*	c.1564delG	p.Ala522Argfs*26	0.5	Dyskeratosis congenita	AR	PR
P37*	AML	39/F	*WRAP53*	c.1564delG	p.Ala522Argfs*26	0.54	Dyskeratosis congenita	AR	CR
P20	AML	25/F	*BLM*	c.320dupT	p.Leu107Phefs*36	0.49	Bloom syndrome	AR	CR
P111	MPN	83/F	*BRCA2*	c.10150C>T	p.Arg3384*	0.5	Fanconi anemia, hereditary breast and ovarian cancer	AR, AD	ND
P136	AML	40/M	*BRCA2*	c.10150C>T	p.Arg3384*	0.52	Fanconi anemia, hereditary breast and ovarian cancer	AR, AD	ND
P13	AML	41/F	*PALB2*	Exon 8 deletion		0.5	Fanconi anemia, hereditary breast and ovarian cancer	AR, AD	CR
P66	MPN	72/M	*PALB2*	c.1240C>T	p.Arg414*	0.4	Fanconi anemia, hereditary breast and ovarian cancer	AR, AD	ND
P126	MPN	59/F	*RAD51*	c.1dupA	p.Met1?	0.49	Fanconi anemia	AR	ND
P69	MPN	54/M	*SBDS*	c.258+2T>C	-	0.46	Shwachman-Diamond syndrome	AR	ND

AD, autosomal dominant; AR, autosomal recessive; BM, bone marrow; CR, complete remission; PR, partial remission; ND, not done

* Confirmed in germline samples

^†^ For all genes, almost only germline mutations are thus far reported in myeloid neoplasms

Among 31 patients with MPN, four (12.9%) were thought to have germline mutations in genes for Fanconi anemia and Shwachman-Diamond syndrome. Contrary to our expectations, older adult patients also had a high frequency of germline mutations.

More than half (8/15; 53.3%) of our cases were mutation carriers of genes for Fanconi anemia, and among the genes, *BRCA2* and *PALB2* are known to be associated with other cancers, including breast and ovarian cancers. One case was a mutation carrier of *ATM*, which is also associated with risks for other solid cancers. These patients could benefit from genetic counseling and preventive monitoring and risk reduction for other cancers.

## Discussion

Owing to the diverse types of genetic abnormalities, conventional workup for AML and other myeloid neoplasms requires a variety of methods, including chromosome analysis, FISH, RT-PCR, real-time PCR, fluorescence PCR and fragment analysis, MLPA, and Sanger sequencing. Recent NGS technologies are able to obtain a great deal of genomic information from a single assay. Moreover, in addition to detection of sequence variations, we believe that a single NGS assay has the potential to replace many conventional assays. By adopting various bioinformatics algorithms, we validated that extensive analyses on NGS data can yield results comparable to many conventional molecular and cytogenetic assays. Our custom NGS panel consists of 215 genes. Although recurrently mutated genes are limited in number, we added a number of genes associated with cancer predisposition to the panel: in WHO diagnostic criteria, more than 40 genes are listed in association with MFS [28]. We composed the large panel in an attempt to maximize the possibility of mutation detection in germline predisposition genes.

*FLT3* ITD is a duplication of a juxtamembrane domain of the *FLT3* gene, and the mutations are reported to range in size from 3 to more than 400 bp. Detection of *FLT3* ITD is essential in the management of AML due to its high impact on prognostic stratification and treatment decision [29]. Standard variant detection tools for NGS data, such as GATK, are usually designed to detect SNVs and thus can miss large size genetic alterations [30]. Therefore, we adopted the Pindel algorithm, which is based on a split-read analysis and optimized specifically for detecting large insertions or deletions [7], and were able to confirm its excellent performance.

Detecting genic or exonic level CNVs is still challenging, and different algorithms have their own strengths and weaknesses [30]. We adopted the ExomeDepth algorithm, which is highly sensitive, even in detecting small deletions or duplications, but produces more false-positives [14]. This was further validated using the DxSeq Analyzer; the variability of read depth in each base position was normalized and illustrated. With visual inspection, CNV calls in regions with high variability among samples in the same batch or without neat signals were assumed more likely to be false-positives. The validity of our approach was further confirmed using MLPA testing. The clinical utility of CNV analysis was evident in detecting *KMT2A* PTD, which is associated with poor prognosis, but its detection is tricky because of the need to amplify common rearrangement breakpoints using RNA samples [31,32]. Without additional testing, we could easily detect *KMT2A* PTD by utilizing computational algorithms (Fig 3). We suspect that patients with CNVs in genes associated with a worse prognosis, such as *TP53*, might also benefit from our assay, the results from which could potential help in reconsidering prognostic stratification and modifying treatment.

Recycling “garbage” data using CopywriteR was another innovative approach [15]. Sequence reads outside target regions have previously been discarded from mutation analysis; however, reanalyzing off-target data could give us a complete view of genome wide copy number status. With this chromosomal CNV analysis, we could identify patients with cryptic losses or gains that were missed by conventional cytogenetics. The analysis could not detect balanced translocations due to an inherent drawback of signal ratio-based methods, as also seen in chromosomal microarrays.

Detecting gene fusion at the DNA level is an attractive option, considering the difficulties in dealing with unstable RNA in conventional RT-PCR. For this purpose, we specifically designed capture probes targeting intronic breakpoints of recurrent translocations and evaluated various algorithms optimized for detecting large structural variations. The detection rate of BreakDancer, a popular algorithm for translocation detection using paired-end mapping method [8], was low. Delly, a combinatorial method combining paired-end mapping and split-read [9], showed better capability in detecting known translocations, although it was not perfect. This may be due to short read lengths and low read depths around certain breakpoints: some intronic areas are difficult to capture due to the presence of repeat elements or variable GC content. This was suggested by a previous study on a small subset of cases with *ALK* or *KMT2A* translocations [33], and we also found a limitation for many recurrent translocations in AML. Thus, one could conclude that short-read DNA sequencing cannot detect gene fusion effectively, but can provide supplementary information to RNA testing.

By observing unexpectedly high frequencies, we could suggest that the existence of germline predisposition mutations might have been underestimated and neglected thus far. This may be due to the absence of available testing methods that could assess a number of responsible genes before the NGS era. The previously reported *DDX41* A500Cfs*9 mutation was found in two cases [26,27]. The gene encodes an RNA helicase thought to function as a tumor suppressor [34,35], and is known as one of the most frequently mutated predisposition gene in myeloid neoplasms [36]. The spectrum of germline mutations in *DDX41* has revealed distinct ethnically associated mutations, the most common of which (D149Gfs*2) has exclusively been found in Caucasians [27,34,35], whereas A500Cfs*9 has only been documented in Asians [26,27], suggesting the mutation derived from a founder mutation. Although cases with acquisition of a somatic mutation in *DDX41*, in addition to germline mutation, have been reported [34,35], no additional mutations in *DDX41* were identified in our patients.

Patients with biallelic mutations in genes for MFS have ben shown to have a higher probability of acquiring hematologic cancers, such as acute leukemia [37,38]. However, the risk of patients with monoallelic mutation in genes for MFS is largely unknown, although increased risk for other solid cancers has been suggested for some genes [39–42]. Our data suggest that monoallelic carriers also have an increased risk for hematologic malignancies, and this might have different clinical implications as well. Although more data would be required to confirm the consequence of these mutations, some patients showed poor treatment responses and multiple relapses. Having a germline mutation in cancer predisposition genes could lead to an altered susceptibility to chemotherapy or a different bone marrow niche environment in stem cell transplantation. Receiving stem cells from HLA-matched siblings that have a chance of carrying the same germline mutation may be another issue of concern, as cases with donor cell leukemia derived from a germline predisposition mutation in stem cell donors have been reported [26,43].

More than half of the cases with germline predisposition mutations had mutations in genes with increased risk for other cancers. Further genetic counseling for these patients and their family members, along with the recommendation for cancer surveillance and a prevention program, is an important issue. For example, patient P35 was a 26-year-old woman with AML and was found to have a heterozygous *PALB2* mutation. *PALB2* is a *BRCA2*-interacting protein needed for the DNA repair function of *BRCA2* [44], and the spectrum of cancers in patients with the biallelic *PALB2* mutation is very similar to those with biallelic *BRCA2* mutation [39]. Because monoallelic mutation of the *PALB2* gene is associated with an approximately six-fold increased risk of breast cancer in female patients [39,45–47], National Comprehensive Cancer Network guidelines recommend an annual mammogram and breast magnetic resonance imaging for mutation carriers of *PALB2* [48]. Accordingly, the patient and her family members received genetic counseling, and breast cancer surveillance was planned after stem cell transplantation.

There are a few limitations in this study. As NGS testing results of matched germline sample were not available, the possibility of incomplete filtering of germline variants cannot be eliminated. Low capture efficiency is another limitation that needs to be improved.

In conclusion, we demonstrated that NGS testing, as a single assay, can be a good supplement for a number of conventional molecular and cytogenetic tests through careful probe design and comprehensive bioinformatics analyses. Furthermore, we found a high frequency of germline mutations in germline predisposition genes in myeloid neoplasms, which suggests a high implication of germline predisposition categories in the new WHO classification.

## Supporting information

S1 Table Genes included in the myeloid NGS panel. (PDF)

S2 Table List of intronic breakpoint regions for translocation detection and deep intronic regions with pathogenic mutations implicated. (PDF)

S3 Table List of high-depth target regions. (PDF)

S4 Table List of variants identified. (PDF)

S5 Table Concordance rates between Sanger sequencing and NGS results. (PDF)

S6 Table The results of chromosomal CNVs and conventional karyotyping. (PDF)
