# Supplementary material for: Targeted next generation sequencing can serve as an alternative to conventional tests in myeloid neoplasms
Source: PLoS One. 2019 Mar 6;14(3):e0212228. doi: 10.1371/journal.pone.0212228 (PMC6402635; doi:10.1371/journal.pone.0212228)
Supplement: S1 Table — (PDF) [file pone.0212228.s001.pdf]

S1 Table. Genes included in the myeloid NGS panel

Official name	Entrez.ID	Disorder (OMIM)
ABCB7	22	Anemia, sideroblastic, with ataxia, 301310 (3), X-linked recessive; Cerebellar ataxia
ABL1	25	Leukemia, Philadelphia chromosome-positive, resistant to imatinib (3)
ACD	65057	?Dyskeratosis congenita, autosomal dominant 6, 616553 (3), Autosomal recessive, Autosomal dominant; ?Dyskeratosis congenita, autosomal recessive 7, 616553 (3), Autosomal recessive, Autosomal dominant; Hoyeraal-Hreidarsson syndrome
ADA	100	Adenosine deaminase deficiency, partial, 102700 (3), Autosomal recessive, Somatic mosaicism; Severe combined immunodeficiency due to ADA deficiency, 102700 (3), Autosomal recessive, Somatic mosaicism; Adenosine deaminase deficiency
AK2	204	Reticular dysgenesis, 267500 (3), Autosomal recessive; Reticular dysgenesia
ANKRD26	22852	Thrombocytopenia 2, 188000 (3), Autosomal dominant; Thrombocytopaenia 2
AP3B1	8546	Hermansky-Pudlak syndrome 2, 608233 (3), Autosomal recessive; Hermansky-Pudlak syndrome
ASXL1	171023	Bohring-Opitz syndrome, 605039 (3), Autosomal dominant; Myelodysplastic syndrome, somatic, 614286 (3); Bohring-Opitz syndrome
ATM	472	Ataxia-telangiectasia, 208900 (3), Autosomal recessive; {Breast cancer, susceptibility to}, 114480 (3), Autosomal dominant; Lymphoma, B-cell non-Hodgkin, somatic (3); Lymphoma, mantle cell, somatic (3); T-cell prolymphocytic leukemia, somatic (3); Ataxia telangiectasia
ATR	545	?Cutaneous telangiectasia and cancer syndrome, familial, 614564 (3), Autosomal dominant; Seckel syndrome 1, 210600 (3), Autosomal recessive; Seckel syndrome
ATRX	546	Mental retardation, X-linked 52, 300504 (2); ATRX syndrome
BCOR	54880	Microphthalmia, syndromic 2, 300166 (3), X-linked dominant; Oculofaciocardiodental syndrome
BCORL1	63035	; Intellectual disability, coarse face & hypotonia
BLM	641	Bloom syndrome, 210900 (3), Autosomal recessive; Bloom syndrome
BRAF	673	Adenocarcinoma of lung, somatic, 211980 (3); Cardiofaciocutaneous syndrome, 115150 (3), Autosomal dominant; Colorectal cancer, somatic (3); LEOPARD syndrome 3, 613707 (3), Autosomal dominant; Melanoma, malignant, somatic (3); Nonsmall cell lung cancer, somatic (3); Noonan syndrome 7, 613706 (3); Cardio-facio-cutaneous syndrome
BRCA1	672	{Breast-ovarian cancer, familial, 1}, 604370 (3), Autosomal dominant, Multifactorial; {Pancreatic cancer, susceptibility to, 4}, 614320 (3); Breast cancer
BRCA2	675	{Breast cancer, male, susceptibility to}, 114480 (3), Autosomal dominant; {Breast-ovarian cancer, familial, 2}, 612555 (3), Autosomal dominant; Fanconi anemia, complementation group D1, 605724 (3), Autosomal recessive; {Glioblastoma 3}, 613029 (3), Autosomal recessive; {Medulloblastoma}, 155255 (3), Autosomal dominant; {Pancreatic cancer 2}, 613347 (3); {Prostate cancer}, 176807 (3), Autosomal dominant; Wilms tumor, 194070 (3), Autosomal dominant, Somatic mutation; Breast cancer, sporadic, protection against
BRCC3	79184	
BRINP3	339479	
BRIP1	83990	Breast cancer, early-onset, 114480 (3), Autosomal dominant; Fanconi anemia, complementation group J, 609054 (3); ; Breast cancer

C15ORF41	84529	Dyserythropoietic anemia, congenital, type Ib, 615631 (3), Autosomal recessive; Congenital dysorythropoietic anaemia type 1
C17orf97	400566	
CALR	811	Myelofibrosis, somatic, 254450 (3); Thrombocythemia, somatic, 187950 (3); Psychosis, association with
CBL	867	?Juvenile myelomonocytic leukemia, 607785 (3), Autosomal dominant, Somatic mutation; Noonan syndrome-like disorder with or without juvenile myelomonocytic leukemia, 613563 (3), Autosomal dominant; Noonan-like syndrome
CBLB	868	; Diabetes, type 1
CBLC	23624	; Reduced LDL cholesterol levels
CDAN1	146059	Dyserythropoietic anemia, congenital, type Ia, 224120 (3), Autosomal recessive; ; Congenital dysorythropoietic anaemia type 1
CDKN2A	1029	Melanoma and neural system tumor syndrome, 155755 (3), Autosomal dominant; {Melanoma, cutaneous malignant, 2}, 155601 (3), Autosomal dominant; Orolaryngeal cancer, multiple (3); Pancreatic cancer/melanoma syndrome, 606719 (3), Autosomal dominant; Melanoma, increased risk, association with
CEBPA	1050	?Leukemia, acute myeloid, 601626 (3), Autosomal dominant; Leukemia, acute myeloid, somatic, 601626 (3); Acute myeloid leukaemia
CREBBP	1387	Rubinstein-Taybi syndrome, 180849 (3), Autosomal dominant; Blue cone monochromacy, 303700 (3), X-linked recessive; Colorblindness, protan, 303900 (3), X-linked; Rubinstein-Taybi syndrome
CSF2RA	1438	Surfactant metabolism dysfunction, pulmonary, 4, 300770 (3); Pulmonary alveolar proteinosis
CSF3R	1441	Neutropenia, severe congenital, 7, autosomal recessive, 617014 (3); Neutropaenia
CTC1	80169	Cerebroretinal microangiopathy with calcifications and cysts, 612199 (3), Autosomal recessive; Cerebroretinal microangiopathy with calcifications & cysts
CTCF	10664	Mental retardation, autosomal dominant 21, 615502 (3), Autosomal dominant; Intellectual disability
CUX1	1523	
CXCR4	7852	Myelokathexis, isolated (3); WHIM syndrome, 193670 (3), Autosomal dominant; WHIM syndrome
DAXX	1616	
DCLRE1C	64421	Omenn syndrome, 603554 (3), Autosomal recessive; Severe combined immunodeficiency, Athabascan type, 602450 (3), Autosomal recessive; Immunodeficiency, severe combined
DDX41	51428	{Myeloproliferative/lymphoproliferative neoplasms, familial (multiple types), susceptibility to}, 616871 (3), Autosomal dominant; Refractory anaemia with excess blasts, type 1
DKC1	1736	Dyskeratosis congenita, X-linked, 305000 (3), X-linked recessive; Dyskeratosis congenita
DNMT1	1786	Cerebellar ataxia, deafness, and narcolepsy, autosomal dominant, 604121 (3), Autosomal dominant; Neuropathy, hereditary sensory, type IE, 614116 (3), Autosomal dominant; Neurological disorder
DNMT3A	1788	Tatton-Brown-Rahman syndrome, 615879 (3), Autosomal dominant; Overgrowth syndrome with intellectual disability
EED	8726	; Ulcerative colitis, susceptibility to

EGFR	1956	Adenocarcinoma of lung, response to tyrosine kinase inhibitor in, 211980 (3), Autosomal recessive; ?Inflammatory skin and bowel disease, neonatal, 2, 616069 (3), Autosomal recessive; Nonsmall cell lung cancer, response to tyrosine kinase inhibitor in, 211980 (3), Autosomal recessive; {Nonsmall cell lung cancer, susceptibility to}, 211980 (3), Autosomal recessive; Glioblastoma, risk, association with
EGLN1	54583	Erythrocytosis, familial, 3, 609820 (3), Autosomal dominant; [Hemoglobin, high altitude adaptation], 609070 (3), Autosomal dominant; Erythrocytosis and paraganglioma
ELANE	1991	Neutropenia, cyclic, 162800 (3), Autosomal dominant; Neutropenia, severe congenital 1, autosomal dominant, 202700 (3), Autosomal dominant; Neutropaenia, congenital
EPAS1	2034	Erythrocytosis, familial, 4, 611783 (3); Polycythaemia with paraganglioma
EPOR	2057	[Erythrocytosis, familial, 1], 133100 (3), Autosomal dominant; Erythrocytosis
ERCC4	2072	Fanconi anemia, complementation group Q, 615272 (3), Autosomal recessive; ?XFE progeroid syndrome, 610965 (3); Xeroderma pigmentosum, group F, 278760 (3), Autosomal recessive; Xeroderma pigmentosum, type F/Cockayne syndrome, 278760 (3), Autosomal recessive; Xeroderma pigmentosum (F)
ETNK1	55500	
ETV6	2120	Leukemia, acute myeloid, somatic, 601626 (3); Thrombocytopenia 5, 616216 (3), Autosomal dominant; Thrombocytopaenia & haematologic malignancy
EZH2	2146	Weaver syndrome, 277590 (3), Autosomal dominant; ; Weaver syndrome
FAM47A	158724	
FANCA	2175	Fanconi anemia, complementation group A, 227650 (3), Autosomal recessive; Fanconi anaemia
FANCB	2187	Fanconi anemia, complementation group B, 300514 (3); Fanconi anaemia
FANCC	2176	Fanconi anemia, complementation group C, 227645 (3), Autosomal recessive; Fanconi anaemia
FANCD2	2177	Fanconi anemia, complementation group D2, 227646 (3), Autosomal recessive; Fanconi anaemia
FANCE	2178	Fanconi anemia, complementation group E, 600901 (3), Autosomal recessive; Esophageal cancer
FANCF	2188	Fanconi anemia, complementation group F, 603467 (3); Fanconi anaemia
FANCG	2189	Fanconi anemia, complementation group G, 614082 (3); Fanconi anaemia
FANCI	55215	Fanconi anemia, complementation group I, 609053 (3); Fanconi anaemia
FANCL	55120	Fanconi anemia, complementation group L, 614083 (3), Autosomal recessive; Fanconi anaemia
FANCM	57697	; Breast cancer, non-BRCA1/BRCA2 related
FAT1	2195	; Facioscapulohumeral dystrophy-like phenotype
FBXW7	55294	
FLRT2	23768	
FLT3	2322	Leukemia, acute lymphoblastic, somatic, 613065 (3); Leukemia, acute myeloid, reduced survival in, somatic, 601626 (3); Leukemia, acute myeloid, somatic, 601626 (3); Myeloid leukaemia, increased risk, association with
G6PC3	92579	Dursun syndrome, 612541 (3), Autosomal recessive; Neutropenia, severe congenital 4, autosomal recessive, 612541 (3), Autosomal recessive; Neutropaenia, congenital

GATA1	2623	Anemia, X-linked, with/without neutropenia and/or platelet abnormalities, 300835 (3), X-linked recessive; Leukemia, megakaryoblastic, with or without Down syndrome, somatic, 190685 (3); Thrombocytopenia with beta-thalassemia, X-linked, 314050 (3), X-linked recessive; Thrombocytopenia, X-linked, with or without dyserythropoietic anemia, 300367 (3), X-linked recessive; Dyserythropoietic anaemia and megakaryocyte dysplasia
GATA2	2624	Emberger syndrome, 614038 (3), Autosomal dominant; Immunodeficiency 21, 614172 (3), Autosomal dominant; {Leukemia, acute myeloid, susceptibility to}, 601626 (3), Autosomal dominant; {Myelodysplastic syndrome, susceptibility to}, 614286 (3); Monocytopaenia & mycobacterial infection syndrome
GFI1	2672	Neutropenia, nonimmune chronic idiopathic, of adults, 607847 (3); Neutropenia, severe congenital 2, autosomal dominant, 613107 (3); Neutropaenia, congenital
GJB3	2707	Deafness, autosomal dominant 2B, 612644 (3), Autosomal dominant; Deafness, autosomal dominant, with peripheral neuropathy (3); Deafness, autosomal recessive (3); Deafness, digenic, GJB2/GJB3, 220290 (3), Autosomal recessive, Digenic dominant; Erythrokeratodermia variabilis et progressiva, 133200 (3), Autosomal recessive, Autosomal dominant; Erythrokeratodermia variabilis
GNAS	2778	ACTH-independent macronodular adrenal hyperplasia, 219080 (3), Isolated cases; Acromegaly, somatic, 102200 (3); McCune-Albright syndrome, somatic, mosaic, 174800 (3); Osseous heteroplasia, progressive, 166350 (3), Autosomal dominant; Pseudohypoparathyroidism Ia, 103580 (3), Autosomal dominant; Pseudohypoparathyroidism Ib, 603233 (3), Autosomal dominant; Pseudohypoparathyroidism Ic, 612462 (3), Autosomal dominant; Pseudopseudohypoparathyroidism, 612463 (3), Autosomal dominant; Albright hereditary osteodystrophy
GNB1	2782	Leukemia, acute lymphoblastic, somatic, 613065 (3); Mental retardation, autosomal dominant 42, 616973 (3), Autosomal dominant; Neurodevelopmental delay, infantile spasms and focal seizures
GPRC5A	9052	; BRCA1-related breast cancer risk, modifier of
HAX1	10456	Neutropenia, severe congenital 3, autosomal recessive, 610738 (3), Autosomal recessive; Kostmann disease
HBB	3043	Delta-beta thalassemia, 141749 (3), Autosomal dominant; Erythremias, beta- (3); Heinz body anemias, beta-, 140700 (3), Autosomal dominant; Hereditary persistence of fetal hemoglobin, 141749 (3), Autosomal dominant; {Malaria, resistance to}, 611162 (3); Methemoglobinemias, beta- (3); Sickle cell anemia, 603903 (3), Autosomal recessive; Thalassemia-beta, dominant inclusion-body, 603902 (3); Thalassemias, beta-, 613985 (3); Thalassaemia beta
HNRNPK	3190	Au-Kline syndrome, 616580 (3), Autosomal dominant; Intellectual disability, facial dysmorphisms and skeletal and connective tissue abnormalities
HRAS	3265	{Bladder cancer, somatic}, 109800 (3); Congenital myopathy with excess of muscle spindles, 218040 (3), Autosomal dominant, Isolated cases; Costello syndrome, 218040 (3), Autosomal dominant, Isolated cases; {Nevus sebaceous or woolly hair nevus, somatic}, 162900 (3); Schimmelpenning-Feuerstein-Mims syndrome, somatic mosaic, 163200 (3); {Spitz nevus or nevus spilus, somatic}, 137550 (3); {Thyroid carcinoma, follicular, somatic}, 188470 (3); Costello syndrome
IDH1	3417	{Glioma, susceptibility to, somatic}, 137800 (3); Osteoarthritis, early-onset
IDH2	3418	D-2-hydroxyglutaric aciduria 2, 613657 (3); D-2-hydroxyglutaric aciduria, type II

IFNG	3458	{AIDS, rapid progression to}, 609423 (3); {Aplastic anemia}, 609135 (3); {Hepatitis C virus, response to therapy of}, 609532 (3); {TSC2 angiomyolipomas, renal, modifier of}, 613254 (3), Autosomal dominant; {Tuberculosis, protection against}, 607948 (3); Hashimoto disease, severity, association with
IKZF1	10320	Immunodeficiency, common variable, 13, 616873 (3), Autosomal dominant
IL2RG	3561	Combined immunodeficiency, X-linked, moderate, 312863 (3), X-linked recessive; Severe combined immunodeficiency, X-linked, 300400 (3), X-linked recessive; Immunodeficiency, severe combined
IRF1	3659	Gastric cancer, somatic, 613659 (3); Myelodysplastic syndrome, preleukemic (3); Myelogenous leukemia, acute (3); Nonsmall cell lung cancer, somatic, 211980 (3); Reduced expression
JAGN1	84522	Neutropenia, severe congenital, 6, autosomal recessive, 616022 (3), Autosomal recessive; Neutropaenia
JAK1	3716	
JAK2	3717	{Budd-Chiari syndrome, somatic}, 600800 (3); Erythrocytosis, somatic, 133100 (3); Leukemia, acute myeloid, somatic, 601626 (3); Myelofibrosis, somatic, 254450 (3); Polycythemia vera, somatic, 263300 (3); Thrombocythemia 3, 614521 (3), Autosomal dominant, Somatic mutation; Thrombocytosis
JAK3	3718	SCID, autosomal recessive, T-negative/B-positive type, 600802 (3), Autosomal recessive; Immunodeficiency, severe combined
KAT6A	7994	Mental retardation, autosomal dominant 32, 616268 (3), Autosomal dominant; Hypotonia, intellectual disability, feeding/oromotor difficulties, microcephaly/craniosynostosis, cardiac & facial defects
KCNA4	3739	
KCNK13	56659	
KDM6A	7403	Kabuki syndrome 2, 300867 (3), X-linked dominant; Renal cancer
KDR	3791	Hemangioma, capillary infantile, somatic, 602089 (3); {Hemangioma, capillary infantile, susceptibility to}, 602089 (3), Autosomal dominant; Coronary heart disease, association with
KIF23	9493	; Intellectual disability and microcephaly
KIT	3815	Gastrointestinal stromal tumor, familial, 606764 (3), Autosomal dominant, Isolated cases; Germ cell tumors, 273300 (3), Somatic mutation; Leukemia, acute myeloid, 601626 (3), Autosomal dominant; Mast cell disease, 154800 (3), Autosomal dominant; Piebaldism, 172800 (3), Autosomal dominant; Mastocytosis
KLF1	10661	Blood group--Lutheran inhibitor, 111150 (3); Dyserythropoietic anemia, congenital, type IV, 613673 (3), Autosomal dominant; [Hereditary persistence of fetal hemoglobin], 613566 (3); Hereditary persistence of foetal haemoglobin
KMT2A	4297	Leukemia, myeloid/lymphoid or mixed-lineage, 159555 (2), Autosomal dominant; Wiedemann-Steiner syndrome, 605130 (3), Autosomal dominant; Epileptic encephalopathy with infantile spasms
KMT2C	58508	; Autism
KRAS	3845	Bladder cancer, somatic, 109800 (3); Breast cancer, somatic, 114480 (3); Cardiofaciocutaneous syndrome 2, 615278 (3); Gastric cancer, somatic, 137215 (3); Leukemia, acute myeloid, 601626 (3), Autosomal dominant; Lung cancer, somatic, 211980 (3); Noonan syndrome 3, 609942 (3); Pancreatic carcinoma, somatic, 260350 (3); RAS-associated autoimmune leukoproliferative disorder, 614470 (3), Autosomal dominant; Schimmelpenning-Feuerstein-Mims syndrome, somatic mosaic, 163200 (3); Lung cancer, risk, association with
LAMB4	22798	; Colorectal cancer

LAMTOR2	28956	Immunodeficiency due to defect in MAPBP-interacting protein, 610798 (3), Autosomal recessive; Primary immunodeficiency disease
LIG4	3981	LIG4 syndrome, 606593 (3); {Multiple myeloma, resistance to}, 254500 (3), Somatic mutation; LIG4 syndrome
LRRC4	64101	; Autism spectrum disorder
LUC7L2	51631	
LYST	1130	Chediak-Higashi syndrome, 214500 (3), Autosomal recessive; Chediak-Higashi syndrome
MAP2K1	5604	Cardiofaciocutaneous syndrome 3, 615279 (3); Cardio-facio-cutaneous syndrome
MCD1	#N/A	
MPL	4352	Myelofibrosis with myeloid metaplasia, somatic, 254450 (3); Thrombocythemia 2, 601977 (3), Autosomal dominant, Somatic mutation; Thrombocytopenia, congenital amegakaryocytic, 604498 (3), Autosomal recessive; Amegakaryocytic thrombocytopaenia, congenital
MRE11A	4361	Ataxia-telangiectasia-like disorder, 604391 (3), Autosomal recessive; Ataxia telangiectasia-like disease
MYC	4609	Burkitt lymphoma, 113970 (3), Isolated cases; Myoclonus, familial cortical, 614937 (3), Autosomal dominant; Colorectal and prostate cancer, association with
MYD88	4615	Macroglobulinemia, Waldenstrom, somatic, 153600 (3); Pyogenic bacterial infections, recurrent, due to MYD88 deficiency, 612260 (3); Reduced function
NBN	4683	Aplastic anemia, 609135 (3); Leukemia, acute lymphoblastic, 613065 (3); Nijmegen breakage syndrome, 251260 (3), Autosomal recessive; Nijmegen breakage syndrome
NCOR2	9612	; Schizophrenia
NF1	4763	Leukemia, juvenile myelomonocytic, 607785 (3), Autosomal dominant, Somatic mutation; Neurofibromatosis, familial spinal, 162210 (3), Autosomal dominant; Neurofibromatosis, type 1, 162200 (3), Autosomal dominant; Neurofibromatosis-Noonan syndrome, 601321 (3), Autosomal dominant; Watson syndrome, 193520 (3), Autosomal dominant; Neurofibromatosis
NHP2	55651	Dyskeratosis congenita, autosomal recessive 2, 613987 (3), Autosomal recessive; Dyskeratosis congenita
NOP10	55505	Dyskeratosis congenita, autosomal recessive 1, 224230 (3), Autosomal recessive; Dyskeratosis congenita, autosomal recessive
NOTCH1	4851	Adams-Oliver syndrome 5, 616028 (3), Autosomal dominant; Aortic valve disease 1, 109730 (3), Autosomal dominant; Aortic valve disease
NPM1	4869	Leukemia, acute myeloid, somatic, 601626 (3)
NRAS	4893	Colorectal cancer, somatic, 114500 (3); Epidermal nevus, somatic, 162900 (3); Melanocytic nevus syndrome, congenital, somatic, 137550 (3); Neurocutaneous melanosis, somatic, 249400 (3); Noonan syndrome 6, 613224 (3), Autosomal dominant; ?RAS-associated autoimmune lymphoproliferative syndrome type IV, somatic, 614470 (3); Schimmelpenning-Feuerstein-Mims syndrome, somatic mosaic, 163200 (3); Thyroid carcinoma, follicular, somatic, 188470 (3); Noonan syndrome
NSD1	64324	Beckwith-Wiedemann syndrome, 130650 (3), Autosomal dominant; Leukemia, acute myeloid, 601626 (1), Autosomal dominant; Sotos syndrome 1, 117550 (3), Autosomal dominant; Sotos syndrome
NTRK3	4916	; Congenital heart defects
OR13H1	347468	
OR8B12	219858	
P2RY2	5029	

PALB2	79728	{Breast cancer, susceptibility to}, 114480 (3), Autosomal dominant; Fanconi anemia, complementation group N, 610832 (3); {Pancreatic cancer, susceptibility to, 3}, 613348 (3); Breast cancer
PARN	5073	Dyskeratosis congenita, autosomal recessive 6, 616353 (3), Autosomal recessive; Pulmonary fibrosis and/or bone marrow failure, telomere-related, 4, 616371 (3), Autosomal dominant; Dyskeratosis congenita
PAX5	5079	{Leukemia, acute lymphoblastic, susceptibility to, 3}, 615545 (3); Acute lymphoblastic leukaemia
PCDHB1	29930	
PDGFRA	5156	Gastrointestinal stromal tumor, somatic, 606764 (3); Hypereosinophilic syndrome, idiopathic, resistant to imatinib, 607685 (3), Isolated cases, Somatic mutation; Cleft palate, isolated
PHF6	84295	Borjeson-Forssman-Lehmann syndrome, 301900 (3), X-linked recessive; Borjeson-Forssman-Lehmann syndrome
PIGA	5277	Multiple congenital anomalies-hypotonia-seizures syndrome 2, 300868 (3), X-linked recessive; Paroxysmal nocturnal hemoglobinuria, somatic, 300818 (3); Cleft palate, neonatal seizures, contractures and central nervous system malformations
PML	5371	Leukemia, acute promyelocytic, PML/RARA type (3); Colon cancer
POT1	25913	{Glioma susceptibility 9}, 616568 (3), Autosomal dominant; {Melanoma, cutaneous malignant, susceptibility to, 10}, 615848 (3), Autosomal dominant; Cardiac angiosarcoma
PRAMEF2	65122	
PRF1	5551	Aplastic anemia, 609135 (3); Hemophagocytic lymphohistiocytosis, familial, 2, 603553 (3), Autosomal recessive; Lymphoma, non-Hodgkin, 605027 (3); Haemophagocytic lymphohistiocytosis, familial
PRPF40B	25766	
PTEN	5728	Bannayan-Riley-Ruvalcaba syndrome, 153480 (3), Autosomal dominant; Cowden syndrome 1, 158350 (3), Autosomal dominant; Endometrial carcinoma, somatic, 608089 (3); {Glioma susceptibility 2}, 613028 (3); Lhermitte-Duclos syndrome, 158350 (3), Autosomal dominant; Macrocephaly/autism syndrome, 605309 (3), Autosomal dominant; Malignant melanoma, somatic, 155600 (3); {Meningioma}, 607174 (3), Autosomal dominant; PTEN hamartoma tumor syndrome (3); {Prostate cancer, somatic}, 176807 (3); Squamous cell carcinoma, head and neck, somatic, 275355 (3); VATER association with macrocephaly and ventriculomegaly, 276950 (3), Autosomal recessive; Cowden disease
PTPN11	5781	LEOPARD syndrome 1, 151100 (3), Autosomal dominant; Leukemia, juvenile myelomonocytic, somatic, 607785 (3); Metachondromatosis, 156250 (3), Autosomal dominant; Noonan syndrome 1, 163950 (3), Autosomal dominant; Noonan syndrome
RAB27A	5873	Griscelli syndrome, type 2, 607624 (3), Autosomal recessive; Griscelli syndrome type 2
RAC1	5879	
RAC2	5880	Neutrophil immunodeficiency syndrome, 608203 (3); Neutrophil immunodeficiency syndrome
RAD21	5885	Cornelia de Lange syndrome 4, 614701 (3), Autosomal dominant; Chronic intestinal pseudo-obstruction
RAD50	10111	Nijmegen breakage syndrome-like disorder, 613078 (3); Breast and/or ovarian cancer
RAD51	5888	{Breast cancer, susceptibility to}, 114480 (3), Autosomal dominant; Mirror movements 2, 614508 (3), Autosomal dominant; Breast cancer, increased risk in BRCA2 carriers, association with

RAD51C	5889	{Breast-ovarian cancer, familial, susceptibility to, 3}, 613399 (3); Fanconi anemia, complementation group O, 613390 (3), Autosomal recessive; Breast and/or ovarian cancer
RAF1	5894	Cardiomyopathy, dilated, 1NN, 615916 (3), Autosomal dominant; LEOPARD syndrome 2, 611554 (3); Noonan syndrome 5, 611553 (3); Cardiomyopathy, dilated
RB1	5925	Bladder cancer, somatic, 109800 (3); Osteosarcoma, somatic, 259500 (3); Retinoblastoma, 180200 (3), Autosomal dominant, Somatic mutation; Retinoblastoma, trilateral, 180200 (3), Autosomal dominant, Somatic mutation; Small cell cancer of the lung, somatic, 182280 (3); Retinoblastoma
RBM8A	9939	Thrombocytopenia-absent radius syndrome, 274000 (3), Autosomal recessive; Reduced promoter activity
RIT1	6016	Noonan syndrome 8, 615355 (3), Autosomal dominant; Noonan syndrome
RMRP	6023	Anauxetic dysplasia, 607095 (3), Autosomal recessive; Cartilage-hair hypoplasia, 250250 (3), Autosomal recessive; Metaphyseal dysplasia without hypotrichosis, 250460 (3), Autosomal recessive; Cartilage-Hair hypoplasia
RNF168	165918	RIDDLE syndrome, 611943 (3), Autosomal recessive; RNF168 deficiency
RPL10	6134	{Autism, susceptibility to, X-linked 5}, 300847 (3); Microcephaly
RPL11	6135	Diamond-Blackfan anemia 7, 612562 (3); Diamond-Blackfan anaemia
RPL15	6138	?Diamond-Blackfan anemia 12, 615550 (3), Autosomal dominant
RPL26	6154	?Diamond-Blackfan anemia 11, 614900 (3), Autosomal dominant; Diamond-Blackfan anaemia
RPL35A	6165	Diamond-Blackfan anemia 5, 612528 (3); Diamond-Blackfan anaemia
RPL5	6125	Diamond-Blackfan anemia 6, 612561 (3); Diamond-Blackfan anaemia
RPS10	6204	Diamond-Blackfan anemia 9, 613308 (3); Diamond-Blackfan anaemia
RPS14	6208	Macrocytic anemia, refractory, due to 5q deletion, somatic, 153550 (3)
RPS15	6209	; Diamond-Blackfan anaemia
RPS17	6218	Diamond-Blackfan anemia 4, 612527 (3)
RPS19	6223	Diamond-Blackfan anemia 1, 105650 (3), Autosomal dominant; Diamond-Blackfan anaemia
RPS24	6229	Diamond-blackfan anemia 3, 610629 (3); Diamond-Blackfan anaemia
RPS26	6231	Diamond-Blackfan anemia 10, 613309 (3), Autosomal dominant; Diamond-Blackfan anaemia
RPS7	6201	Diamond-Blackfan anemia 8, 612563 (3); Diamond-Blackfan anaemia
RTEL1	51750	Dyskeratosis congenita, autosomal dominant 4, 615190 (3), Autosomal recessive, Autosomal dominant; Dyskeratosis congenita, autosomal recessive 5, 615190 (3), Autosomal recessive, Autosomal dominant; Pulmonary fibrosis and/or bone marrow failure, telomere-related, 3, 616373 (3), Autosomal dominant; Dyskeratosis congenita
RUNX1	861	Leukemia, acute myeloid, 601626 (3), Autosomal dominant; Platelet disorder, familial, with associated myeloid malignancy, 601399 (3), Autosomal dominant; Platelet disorder, familial
SAXO2	283726	
SBDS	51119	{Aplastic anemia, susceptibility to}, 609135 (3); Shwachman-Diamond syndrome, 260400 (3), Autosomal recessive; Shwachman-Diamond syndrome
SBF2	81846	Charcot-Marie-Tooth disease, type 4B2, 604563 (3), Autosomal recessive; Charcot-Marie-Tooth disease 4b2
SEC23B	10483	Cowden syndrome 7, 616858 (3), Autosomal dominant; Dyserythropoietic anemia, congenital, type II, 224100 (3), Autosomal recessive; Anaemia, dyserythropoietic congenital, type II

SETBP1	26040	Mental retardation, autosomal dominant 29, 616078 (3), Autosomal dominant; Schinzel-Giedion midface retraction syndrome, 269150 (3), Autosomal dominant; Developmental delay
SF1	7536	
SF3A1	10291	
SF3B1	23451	Myelodysplastic syndrome, somatic, 614286 (3); Myelodysplastic syndrome, somatic, 614286 (3); Miller-Dieker lissencephaly syndrome, 247200 (4), Autosomal dominant; ; Bohring-Opitz syndrome, 605039 (3), Autosomal dominant; Myelodysplastic syndrome, somatic, 614286 (3)
SH2B3	10019	Erythrocytosis, somatic, 133100 (3); Myelofibrosis, somatic, 254450 (3); Thrombocythemia, somatic, 187950 (3); Celiac disease, association with
SH2D1A	4068	Lymphoproliferative syndrome, X-linked, 1, 308240 (3), X-linked recessive; Lymphoproliferative syndrome, X-linked
SLC37A4	2542	Glycogen storage disease Ib, 232220 (3), Autosomal recessive; Glycogen storage disease Ic, 232240 (3), Autosomal recessive
SLX4	84464	Fanconi anemia, complementation group P, 613951 (3), Autosomal recessive; Breast and/or ovarian cancer
SMC1A	8243	Cornelia de Lange syndrome 2, 300590 (3), X-linked dominant; Cornelia de Lange syndrome
SMC3	9126	Cornelia de Lange syndrome 3, 610759 (3), Autosomal dominant; Cornelia de Lange syndrome
SOS1	6654	?Fibromatosis, gingival, 1, 135300 (3), Autosomal dominant; Noonan syndrome 4, 610733 (3), Autosomal dominant; Anemia, X-linked, with/without neutropenia and/or platelet abnormalities, 300835 (3), X-linked recessive; Leukemia, megakaryoblastic, with or without Down syndrome, somatic, 190685 (3); Thrombocytopenia with beta-thalassemia, X-linked, 314050 (3), X-linked recessive; Thrombocytopenia, X-linked, with or without dyserythropoietic anemia, 300367 (3), X-linked recessive; Noonan syndrome
SRP72	6731	Bone marrow failure syndrome 1, 614675 (3), Autosomal dominant; Aplastic anaemia / myelodysplasia
SRSF2	6427	; RNA binding affinity
STAG1	10274	
STAG2	10735	; Diaphragmatic hernia, congenital
STX11	8676	Hemophagocytic lymphohistiocytosis, familial, 4, 603552 (3), Autosomal recessive; Haemophagocytic lymphohistiocytosis, familial
STXBP2	6813	Hemophagocytic lymphohistiocytosis, familial, 5, 613101 (3); Hemophagocytic lymphohistiocytosis
SUZ12	23512	
SYNE1	23345	Emery-Dreifuss muscular dystrophy 4, autosomal dominant, 612998 (3), Autosomal dominant; Spinocerebellar ataxia, autosomal recessive 8, 610743 (3), Autosomal recessive; Arthrogryposis
TAZ	6901	Barth syndrome, 302060 (3), X-linked recessive; Barth syndrome
TCIRG1	10312	Osteopetrosis, autosomal recessive 1, 259700 (3), Autosomal recessive; Osteopetrosis, autosomal recessive
TERC	7012	{Aplastic anemia}, 614743 (3), Autosomal dominant; Dyskeratosis congenita, autosomal dominant 1, 127550 (3), Autosomal dominant; {Pulmonary fibrosis, idiopathic, susceptibility to}, 614743 (3), Autosomal dominant; Shorter telomere length, association with

TERT	7015	{Dyskeratosis congenita, autosomal dominant 2}, 613989 (3), Autosomal recessive, Autosomal dominant; {Dyskeratosis congenita, autosomal recessive 4}, 613989 (3), Autosomal recessive, Autosomal dominant; {Leukemia, acute myeloid}, 601626 (3), Autosomal dominant; {Melanoma, cutaneous malignant, 9}, 615134 (3); {Pulmonary fibrosis and/or bone marrow failure, telomere-related, 1}, 614742 (3), Autosomal dominant; Dyskeratosis congenita
TET1	80312	; Autism
TET2	54790	Myelodysplastic syndrome, somatic, 614286 (3); Prostate cancer
TINF2	26277	Dyskeratosis congenita, autosomal dominant 3, 613990 (3), Autosomal dominant; Revesz syndrome, 268130 (3), Autosomal dominant; Dyskeratosis congenita
TP53	7157	Adrenal cortical carcinoma, 202300 (3), Autosomal recessive; {Basal cell carcinoma 7}, 614740 (3); Breast cancer, 114480 (3), Autosomal dominant; Choroid plexus papilloma, 260500 (3), Autosomal dominant; Colorectal cancer, 114500 (3), Autosomal dominant; {Glioma susceptibility 1}, 137800 (3), Autosomal dominant, Somatic mutation; Hepatocellular carcinoma, 114550 (3), Somatic mutation; Li-Fraumeni syndrome, 151623 (3), Autosomal dominant; Nasopharyngeal carcinoma, 607107 (3); Osteosarcoma, 259500 (3), Autosomal recessive; Pancreatic cancer, 260350 (3), Autosomal dominant, Somatic mutation, Multifactorial; Basal cell carcinoma, increased risk, association with
TSR2	90121	?Diamond-Blackfan anemia 14 with mandibulofacial dysostosis, 300946 (3), X-linked recessive; Diamond-Blackfan anaemia with mandibulofacial dystostosis
TUBA3C	7278	
U2AF1	7307	
U2AF2	11338	
UBE2T	29089	Fanconi anemia, complementation group T, 616435 (3), Autosomal recessive; Breast cancer
UNC13D	201294	Hemophagocytic lymphohistiocytosis, familial, 3, 608898 (3); Haemophagocytic lymphohistiocytosis, familial
USB1	79650	Poikiloderma with neutropenia, 604173 (3), Autosomal recessive; Poikiloderma, clericuzio-type with neutropaenia
VHL	7428	Erythrocytosis, familial, 2, 263400 (3), Autosomal recessive; Hemangioblastoma, cerebellar, somatic (3); Pheochromocytoma, 171300 (3), Autosomal dominant; Renal cell carcinoma, somatic, 144700 (3); von Hippel-Lindau syndrome, 193300 (3), Autosomal dominant; Von Hippel-Lindau syndrome
VPS13B	157680	Cohen syndrome, 216550 (3), Autosomal recessive; Cohen syndrome
VPS45	11311	Neutropenia, severe congenital, 5, autosomal recessive, 615285 (3), Autosomal recessive; Neutropaenia, thrombasthenia & myelofibrosis of infancy
WAS	7454	Neutropenia, severe congenital, X-linked, 300299 (3), X-linked recessive; Thrombocytopenia, X-linked, 313900 (3), X-linked recessive; Thrombocytopenia, X-linked, intermittent, 313900 (3), X-linked recessive; Wiskott-Aldrich syndrome, 301000 (3), X-linked recessive; Wiskott-Aldrich syndrome
WRAP53	55135	Dyskeratosis congenita, autosomal recessive 3, 613988 (3), Autosomal recessive; Dyskeratosis congenita

WT1	7490	Denys-Drash syndrome, 194080 (3), Autosomal dominant, Somatic mutation; Frasier syndrome, 136680 (3), Autosomal dominant, Somatic mutation; Meacham syndrome, 608978 (3); Mesothelioma, somatic, 156240 (3); Nephrotic syndrome, type 4, 256370 (3), Autosomal dominant; Wilms tumor, type 1, 194070 (3), Autosomal dominant, Somatic mutation; Frasier syndrome
XIAP	331	Lymphoproliferative syndrome, X-linked, 2, 300635 (3); XIAP deficiency
XRCC2	7516	; Breast cancer
ZRSR2	8233	; Intellectual disability, X-linked
