# Supplementary material for: Targeted next generation sequencing can serve as an alternative to conventional tests in myeloid neoplasms
Source: PLoS One. 2019 Mar 6;14(3):e0212228. doi: 10.1371/journal.pone.0212228 (PMC6402635; doi:10.1371/journal.pone.0212228)
Supplement: S2 Table — (PDF) [file pone.0212228.s002.pdf]

S2 Table. List of intronic breakpoint regions for translocation detection and deep intronic regions with pathogenic mutations implicated

gene	Target	Type	chrom	start	end	size (bp)
KMT2A	introns 8-13 (MLL-PTD, MLL translocation)	intronic breakpoints	11	118352807	118360844	8038
TERT	promoter	deep intron mutation site (HGMD)	5	1295161	1295250	90
UNC13D	intron 1	deep intron mutation site (HGMD)	17	73839908	73839908	1
ANKRD26	5'UTR	deep intron mutation site (HGMD)	10	27389256	27389427	172
GATA2	intron 5 enhancer	deep intron mutation site (HGMD)	3	128202118	128202197	80
TERC	all noncoding region	deep intron mutation site (ClinVar)	3	169482398	169482848	451
PDGFRB	introns 8-13	intronic breakpoints	5	149503923	149511542	7620
PDGFRA	intron 11	intronic breakpoints	4	55140698	55141140	443
FGFR1	intron 9	intronic breakpoints	8	38275746	38277253	1508
JAK2	introns 7-19 (PCM1/JAK2, ETV6/JAK2)	intronic breakpoints	9	5054884	5081725	26842
RUNX1	intron 6 (RUNX1/RUNX1T1)	intronic breakpoints	21	36206898	36231771	24874
RARA	intron 2 (PML/RARA)	intronic breakpoints	17	38487448	38504716	17269
MYH11	introns 27-33 (CBFB/MYH11)	intronic breakpoints	16	15814008	15826565	12558
NUP214	intron 17 (DEK/NUP214)	intronic breakpoints	9	134027123	134034873	7751
RBM15	RBM15/MKL1	intronic breakpoints	1	110881945	110889303	7359
RBM8A	NM_005105:c.-21G>A	deep intron mutation site (ClinVar)	1	145507646	145507646	1
RBM8A	NM_005105:c.67+32G>C	deep intron mutation site (ClinVar)	1	145507765	145507765	1
RBM8A	NM_005105:c.67+93A>T	deep intron mutation site (ClinVar)	1	145507826	145507826	1
DCLRE1C	NM_001033855:c.973-1777G>C	deep intron mutation site (HGMD)	10	14966845	14966845	1
ANKRD26	NM_014915:c.-134G>A	deep intron mutation site (ClinVar)	10	27389389	27389389	1
PTEN	NM_000314:c.-861G>T	deep intron mutation site (ClinVar)	10	89623365	89623365	1
PTEN	NM_000314:c.-764G>A	deep intron mutation site (ClinVar)	10	89623462	89623462	1
PTEN	NM_000314:c.254-21G>C	deep intron mutation site (HGMD)	10	89692749	89692749	1
ATM	NM_000051:c.1236-404C>T	deep intron mutation site (HGMD)	11	108121024	108121024	1
ATM	NM_000051:c.2639-384A>G	deep intron mutation site (HGMD)	11	108138753	108138753	1
ATM	NM_000051:c.2839-581_2839-578delGTAA	deep intron mutation site (ClinVar)	11	108141210	108141210	1
ATM	NM_000051:c.3994-159A>G	deep intron mutation site (HGMD)	11	108158168	108158168	1
ATM	NM_000051:c.5763-1050A>G	deep intron mutation site (HGMD)	11	108179837	108179837	1
HBB	NM_000518:c.*110_*114delTAAAA	deep intron mutation site (ClinVar)	11	5246714	5246714	1
HBB	NM_000518:c.*113A>G	deep intron mutation site (ClinVar)	11	5246715	5246715	1
HBB	NM_000518:c.*112A>G	deep intron mutation site (ClinVar)	11	5246716	5246716	1
HBB	NM_000518:c.*111A>G	deep intron mutation site (ClinVar)	11	5246717	5246717	1
HBB	NM_000518:c.*110T>C	deep intron mutation site (ClinVar)	11	5246718	5246718	1
HBB	NM_000518:c.316-90A>G	deep intron mutation site (HGMD)	11	5247046	5247046	1

HBB	NM_000518:c.316-106C>G	deep intron mutation site (ClinVar)	11	5247062	5247062	1
HBB	NM_000518:c.316-125A>G	deep intron mutation site (HGMD)	11	5247081	5247081	1
HBB	NM_000518:c.316-146T>G	deep intron mutation site (ClinVar)	11	5247102	5247102	1
HBB	NM_000518:c.316-197C>T	deep intron mutation site (ClinVar)	11	5247153	5247153	1
HBB	NM_000518:c.316-238C>T	deep intron mutation site (HGMD)	11	5247194	5247194	1
HBB	NM_000518:c.316-260T>C	deep intron mutation site (HGMD)	11	5247216	5247216	1
HBB	NM_000518:c.93-21G>A	deep intron mutation site (ClinVar)	11	5248050	5248050	1
HBB	NM_000518:c.93-23T>C	deep intron mutation site (HGMD)	11	5248052	5248052	1
HBB	NM_000518:c.-50A>C	deep intron mutation site (ClinVar)	11	5248301	5248301	1
HBB	NM_000518:c.-78A>G	deep intron mutation site (ClinVar)	11	5248329	5248329	1
HBB	NM_000518:c.-50-29A>G	deep intron mutation site (ClinVar)	11	5248330	5248330	1
HBB	NM_000518:c.-80T>A	deep intron mutation site (ClinVar)	11	5248331	5248331	1
HBB	NM_000518:c.-81A>G	deep intron mutation site (ClinVar)	11	5248332	5248332	1
HBB	NM_000518:c.-82C>A	deep intron mutation site (ClinVar)	11	5248333	5248333	1
HBB	NM_000518:c.-136C>T	deep intron mutation site (ClinVar)	11	5248387	5248387	1
HBB	NM_000518:c.-137C>T	deep intron mutation site (ClinVar)	11	5248388	5248388	1
HBB	NM_000518:c.-50-88C>T	deep intron mutation site (ClinVar)	11	5248389	5248389	1
HBB	NM_000518:c.-140C>T	deep intron mutation site (ClinVar)	11	5248391	5248391	1
HBB	NM_000518:c.-50-92C>T	deep intron mutation site (ClinVar)	11	5248393	5248393	1
HBB	NM_000518:c.-50-101C>T	deep intron mutation site (ClinVar)	11	5248402	5248402	1
TCIRG1	NM_006019:c.1887+132T>C	deep intron mutation site (HGMD)	11	67816893	67816893	1
TCIRG1	NM_006019:c.1887+142T>A	deep intron mutation site (HGMD)	11	67816903	67816903	1
TCIRG1	NM_006019:c.1887+146G>A	deep intron mutation site (HGMD)	11	67816907	67816907	1
TCIRG1	NM_006019:c.1887+149C>T	deep intron mutation site (HGMD)	11	67816910	67816910	1
PTPN11	NM_002834:c.934-59T>A	deep intron mutation site (HGMD)	12	112915602	112915602	1
PTPN11	NM_080601:c.*2403A>G	deep intron mutation site (ClinVar)	12	112926840	112926840	1
PTPN11	NM_080601:c.*2414C>G	deep intron mutation site (ClinVar)	12	112926851	112926851	1
PTPN11	NM_080601:c.*2445G>A	deep intron mutation site (ClinVar)	12	112926882	112926882	1
PTPN11	NM_080601:c.*2451G>A	deep intron mutation site (ClinVar)	12	112926888	112926888	1
KRAS	NM_033360:c.*22C>G	deep intron mutation site (ClinVar)	12	25362828	25362828	1
BRCA2	NM_000059:c.6937+594T>G	deep intron mutation site (HGMD)	13	32919384	32919384	1
BRCA2	NM_000059:c.7008-62A>G	deep intron mutation site (HGMD)	13	32928936	32928936	1
BRCA2	NM_000059:c.9502-28A>G	deep intron mutation site (HGMD)	13	32971007	32971007	1
RB1	NM_000321:c.-198G>A	deep intron mutation site (ClinVar)	13	48877851	48877851	1
RB1	NM_000321:c.-189G>T	deep intron mutation site (ClinVar)	13	48877860	48877860	1
RB1	NM_000321:c.861+828T>G	deep intron mutation site (HGMD)	13	48937921	48937921	1
RB1	NM_000321:c.1215+63T>G	deep intron mutation site (HGMD)	13	48947691	48947691	1

RB1	NM_000321:c.2490-1398A>G	deep intron mutation site (ClinVar)	13	49046098	49046098	1
RB1	NM_000321:c.2490-26A>C	deep intron mutation site (HGMD)	13	49047470	49047470	1
FANCI	NM_001113378:c.1583+142C>T	deep intron mutation site (HGMD)	15	89825208	89825208	1
FANCA	NM_000135:c.3239+82T>G	deep intron mutation site (HGMD)	16	89816056	89816056	1
FANCA	NM_000135:c.2982-192A>G	deep intron mutation site (HGMD)	16	89818822	89818822	1
FANCA	NM_000135:c.2778+83C>G	deep intron mutation site (HGMD)	16	89831215	89831215	1
FANCA	NM_000135:c.2504+134A>G	deep intron mutation site (HGMD)	16	89836111	89836111	1
FANCA	NM_000135:c.2223-138A>G	deep intron mutation site (HGMD)	16	89836805	89836805	1
FANCA	NM_000135:c.893+920C>A	deep intron mutation site (HGMD)	16	89864654	89864654	1
NF1	NM_000267:c.61-7486G>T	deep intron mutation site (HGMD)	17	29475515	29475515	1
NF1	NM_000267:c.288+2025T>G	deep intron mutation site (HGMD)	17	29488136	29488136	1
NF1	NM_000267:c.888+651T>A	deep intron mutation site (HGMD)	17	29510334	29510334	1
NF1	NM_000267:c.888+744A>G	deep intron mutation site (HGMD)	17	29510427	29510427	1
NF1	NM_000267:c.1062+113A>G	deep intron mutation site (HGMD)	17	29527726	29527726	1
NF1	NM_000267:c.1260+1604A>G	deep intron mutation site (HGMD)	17	29530107	29530107	1
NF1	NM_000267:c.1393-592A>G	deep intron mutation site (HGMD)	17	29540877	29540877	1
NF1	NM_000267:c.1527+1159C>T	deep intron mutation site (HGMD)	17	29542762	29542762	1
NF1	NM_000267:c.1642-449A>G	deep intron mutation site (HGMD)	17	29548419	29548419	1
NF1	NM_000267:c.1721+542A>G	deep intron mutation site (HGMD)	17	29549489	29549489	1
NF1	NM_000267:c.3198-314G>A	deep intron mutation site (HGMD)	17	29558777	29558777	1
NF1	NM_000267:c.3974+260T>G	deep intron mutation site (HGMD)	17	29563299	29563299	1
NF1	NM_000267:c.4110+945A>G	deep intron mutation site (HGMD)	17	29577082	29577082	1
NF1	NM_001042492:c.4173+278A>G	deep intron mutation site (HGMD)	17	29580296	29580296	1
NF1	NM_000267:c.5749+332A>G	deep intron mutation site (ClinVar)	17	29657848	29657848	1
NF1	NM_000267:c.5750-279A>G	deep intron mutation site (HGMD)	17	29661577	29661577	1
NF1	NM_000267:c.7908-321C>G	deep intron mutation site (HGMD)	17	29685177	29685177	1
NF1	NM_000267:c.8050+25A>T	deep intron mutation site (HGMD)	17	29685665	29685665	1
BRCA1	NM_007294:c.*102_*105delCTGT	deep intron mutation site (ClinVar)	17	41197590	41197590	1
BRCA1	NM_007294:c.5468-40T>A	deep intron mutation site (ClinVar)	17	41197859	41197859	1
BRCA1	NM_007294:c.4358-2786G>A	deep intron mutation site (ClinVar)	17	41231417	41231417	1
BRIP1	NM_032043:c.1629-498A>T	deep intron mutation site (HGMD)	17	59858864	59858864	1
UNC13D	NM_199242:c.118-308C>T	deep intron mutation site (HGMD)	17	73839908	73839908	1
TP53	NM_000546:c.673-39G>A	deep intron mutation site (HGMD)	17	7577647	7577647	1
TP53	NM_000546:c.97-28T>A	deep intron mutation site (HGMD)	17	7579618	7579618	1
FANCL	NM_018062:c.375-2033C>G	deep intron mutation site (HGMD)	2	58433394	58433394	1
SEC23B	NM_006363:c.221+31A>G	deep intron mutation site (HGMD)	20	18491731	18491731	1
SEC23B	NM_006363:c.221+163A>G	deep intron mutation site (HGMD)	20	18491863	18491863	1

SEC23B	NM_006363:c.222-78C>T	deep intron mutation site (HGMD)	20	18492791	18492791	1
SEC23B	NM_006363:c.1743+168A>G	deep intron mutation site (HGMD)	20	18526845	18526845	1
ADA	NM_000022:c.976-34G>A	deep intron mutation site (HGMD)	20	43249076	43249076	1
FANCD2	NM_033084:c.696-121C>G	deep intron mutation site (HGMD)	3	10083186	10083186	1
VHL	NM_000551:c.-75_-55del21	deep intron mutation site (ClinVar)	3	10183457	10183457	1
GATA2	NM_032638:c.1017+572C>T	deep intron mutation site (HGMD)	3	128202131	128202131	1
ATR	NM_001184:c.6897+464C>G	deep intron mutation site (ClinVar)	3	142184702	142184702	1
CDKN2A	NM_000077:c.458-105A>G	deep intron mutation site (HGMD)	9	21968346	21968346	1
CDKN2A	NM_000077:c.151-1104C>G	deep intron mutation site (HGMD)	9	21972311	21972311	1
CDKN2A	NM_000077:c.150+1104C>A	deep intron mutation site (HGMD)	9	21973573	21973573	1
CDKN2A	NM_000077:c.-34G>T	deep intron mutation site (ClinVar)	9	21974860	21974860	1
TAZ	NM_000116:c.284+110G>A	deep intron mutation site (HGMD)	X	153641699	153641699	1
TAZ	NM_181312:c.541+122G>A	deep intron mutation site (ClinVar)	X	153648084	153648084	1
