# Supplementary material for: Targeted next generation sequencing can serve as an alternative to conventional tests in myeloid neoplasms
Source: PLoS One. 2019 Mar 6;14(3):e0212228. doi: 10.1371/journal.pone.0212228 (PMC6402635; doi:10.1371/journal.pone.0212228)
Supplement: S3 Table — (PDF) [file pone.0212228.s003.pdf]

S3 Table. List of high-depth target regions

Gene	Region
CSF3R	p.T618
MPL	p.W515
KRAS	codon 12-13,and 61
NRAS	codon 12-13,and 61
JAK2	p.V617 and exon 12
FLT3	exon 14-15 and p.D835
IDH1	exon 4
IDH2	exon 4
CALR	exon 9
SF3B1	exon 14-16
ASXL1	exon 12
NPM1	exon 11
KIT	exon 2,8-11,13, and 17
BRAF	p.V600
WT1	exon 7 and 9
TP53	all coding exons
CEBPA	all coding exons
DNMT3A	all coding exons
