# Supplementary material for: Targeted next generation sequencing can serve as an alternative to conventional tests in myeloid neoplasms
Source: PLoS One. 2019 Mar 6;14(3):e0212228. doi: 10.1371/journal.pone.0212228 (PMC6402635; doi:10.1371/journal.pone.0212228)
Supplement: S4 Table — (PDF) [file pone.0212228.s004.pdf]

S4 Table. List of variants identified

ID	Disease	Gene	position		VAF
1	AML	*DNMT3A*	c.2644C>T	p.Arg882Cys	0.47
1	AML	*NPM1*	c.860_863dupTCTG		0.36
1	AML	*KMT2A*	c.7443-1G>T		0.18
2	AML	*DNMT3A*	c.2645G>A	p.Arg882His	0.43
2	AML	*IDH2*	c.515G>A	p.Arg172Lys	0.48
3	AML	*ASXL1*	c.2385delC	p.Trp796GlyfsTer22	0.32
3	AML	*IDH1*	c.394C>T	p.Arg132Cys	0.17
3	AML	*STAG2*	c.768delT	p.Ile256MetfsTer9	0.33
4	AML	*DNMT3A*	c.1851+1G>A		0.36
4	AML	*IDH1*	c.394C>T	p.Arg132Cys	0.36
5	AML	*ASXL1*	c.1934dupG	p.Gly646TrpfsTer12	0.39
5	AML	*CEBPA*	c.934C>T	p.Gln312Ter	0.5
5	AML	*CEBPA*	c.107delG	p.Gly36AlafsTer124	0.51
5	AML	*TET2*	c.4030dupG	p.Ala1344GlyfsTer3	0.46
5	AML	*ATM*	c.5288_5289insGA	p.Tyr1763Ter	0.52
5	AML	*KMT2A*	c.7443-1G>T		0.2
6	AML	*ASXL1*	c.1900_1922del	p.Glu635ArgfsTer15	0.24
6	AML	*IDH2*	c.419G>A	p.Arg140Gln	0.48
6	AML	*RUNX1*	c.1174C>T	p.Gln392Ter	0.88
6	AML	*STAG2*	c.394dupA	p.Thr132AsnfsTer6	0.96
6	AML	*STAG2*	c.1907dupA	p.Tyr636Ter	0.11
6	AML	*CREBBP*	c.5301dupA	p.Arg1768ThrfsTer198	0.43
7	AML	*DNMT3A*	c.1979dupA	p.Tyr660Ter	0.14
7	AML	*WT1*	c.1108_1109insT	p.Arg370LeufsTer15	0.14
7	AML	*NPM1*	c.860_863dupTCTG	p.Trp288CysfsTer12	0.34
8	AML	*DNMT3A*	c.2645G>A	p.Arg882His	0.41
8	AML	*NPM1*	c.860_863dupTCTG	p.Trp288CysfsTer12	0.34
9	AML	*WT1*	c.353C>A	p.Ser118Ter	0.56
9	AML	*KDM6A*	c.3338_3342delTATCA	p.Val1113GlyfsTer36	0.49
9	AML	*PHF6*	c.279_291delTTGTGATGTGAAA	p.Cys94HisfsTer45	0.44
9	AML	*SH2B3*	c.1038dupG	p.Leu347AlafsTer38	0.86
9	AML	*SUZ12*	c.1023+1G>A		0.32
10	AML	*NPM1*	c.860_863dupTCTG		0.37
10	AML	*TET2*	c.3500+2T>C		0.44
11	AML	*NRAS*	c.35G>A	p.Gly12Asp	0.11
11	AML	*EZH2*	c.684dupC	p.Ser229LeufsTer6	0.37
12	AML	*WT1*	c.1394A>G	p.His465Arg	0.59
12	AML	*NRAS*	c.35G>A	p.Gly12Asp	0.36
13	AML	*RNF168*	c.225_226insATTCT	p.Leu76IlefsTer6	0.25
15	AML	*ASXL1*	c.1720-1G>T		0.49
15	AML	*TET2*	c.1299dupA	p.His434ThrfsTer9	0.36
15	AML	*SH2B3*	c.951_954dupGCAT	p.Ile319AlafsTer9	0.39
15	AML	*CUX1*	c.2379delG	p.Leu794TrpfsTer14	0.16
16	AML	*IDH2*	c.419G>A	p.Arg140Gln	0.45
16	AML	*NPM1*	c.860_863dupTCTG		0.35
16	AML	*PTPN11*	c.179G>T	p.Gly60Val	0.33
17	AML	*DNMT3A*	c.2645G>A	p.Arg882His	0.39
17	AML	*FLT3-TKD*	c.2503G>C	D835H	0.4

17	AML	*MPL*	c.1069C>T	p.Arg357Ter	0.39
20	AML	*BLM*	c.320dupT	p.Leu107PhefsTer36	0.49
22	AML	*RUNX1*	c.893dupC	p.Ala299SerfsTer301	0.4
23	AML	*BCOR*	c.1970dupA	p.Tyr657Ter	0.82
24	AML	*CEBPA*	c.322_329dupTACCCGGG	p.Ala111ThrfsTer52	0.31
24	AML	*VPS13B*	c.762+2T>G		0.59
25	AML	*CEBPA*	c.209dup	p.Ala71GlyfsTer37	0.46
25	AML	*CEBPA*	c.917_934delGCAACGTGGAGACG CAGC	p.Arg306_Gln311del	0.25
26	AML	*NRAS*	c.35G>C	p.Gly12Ala	0.16
27	AML	*KIT*	c.2466T>A	p.Asp882Lys	0.43
27	AML	*KDM6A*	c.3140delT	p.Leu1047Ter	0.4
27	AML	*SEC23B*	c.1507C>T	p.Arg503Ter	0.48
28	AML	*LAMB4*	c.3267dupT	p.Ser1090Ter	0.43
30	AML	*ASXL1*	c.1934dupG	p.Gly646TrpfsTer12	0.32
30	MDS	*FANCA*	c.1A>T	p.Met1?	0.46
31	AML	*CEBPA*	c.68dupC	p.His24AlafsTer84	0.47
31	AML	*CEBPA*	c.907_909dupGCC	p.Ala303dup	0.45
32	AML	*CEBPA*	c.241_248delCTGTTCCA		0.2
32	AML	*CEBPA*	c.929_931delCGC		0.28
32	AML	*CEBPA*	c.925_927delGAG		0.29
32	AML	*TET2*	c.3247C>T	p.Gln1083Ter	0.41
32	AML	*KMT2C*	c.8390dupA	p.Glu2798GlyfsTer11	0.38
34	AML	*KIT*	c.2466T>A	p.Asp882Lys	0.38
34	AML	*WRAP53*	c.1564delG	p.Ala522ArgfsTer26	0.5
35	AML	*WT1*	c.1105delC	p.Arg369AspfsTer6	0.35
35	AML	*WT1*	c.1107_1108insGG	p.Arg370GlyfsTer6	0.36
35	AML	*WT1*	c.1101_1102insA	p.Val368SerfsTer17	0.35
35	AML	*PTPN11*	c.172A>T	p.Asn58Tyr	0.25
35	AML	*PALB2*	c.1011_1015delACCAG	p.Leu337PhefsTer3	0.44
37	AML	*CEBPA*	c.759_763delCAAGG		0.34
37	AML	*WT1*	c.1105C>T	p.Arg369Ter	0.51
37	AML	*WRAP53*	c.1564delG	p.Ala522ArgfsTer26	0.54
37	AML	*MPL*	c.972delC	p.Arg325GlufsTer44	0.53
38	AML	*PTPN11*	c.226G>A	p.Glu76Lys	0.41
39	AML	*NPM1*	c.860_863dupTCTG		0.21
39	AML	*WT1*	c.1142_1143insAA	p.Ala382ArgfsTer68	0.3
39	AML	*NRAS*	c.38G>A	p.Gly13Asp	0.19
40	AML	*DNMT3A*	c.2645G>A	p.Arg882His	0.38
40	AML	*PHF6*	c.820C>T	p.Arg274Ter	0.12
40	AML	*NF1*	c.2325+1G>C		0.21
41	AML	*ASXL1*	c.2439delC	p.Ile814PhefsTer4	0.47
41	AML	*IDH2*	c.419G>A	p.Arg140Gln	0.29
41	AML	*RUNX1*	c.292delC	p.Leu98SerfsTer24	0.65
43	AML	*FLT3-TKD*	c.2503G>T	D835Y	0.44
44	AML	*KRAS*	c.34G>T	G12C	0.3
44	AML	*FBXW7*	c.585-1G>T		0.19
45	AML	*NRAS*	c.35G>T	p.Gly12Val	0.44
47	AML	*NRAS*	c.35G>A	p.Gly12Asp	0.2
49	AML	*ASXL1*	c.1934dupG	p.Gly646TrpfsTer12 (e13	0.28
49	AML	*TP53*	c.949delC	p.Gln317SerfsTer28	0.29

52	MDS	*TP53*	c.372C>A	p.Cys124Ter	0.38
52	MDS	*LUC7L2*	c.70C>T	p.Arg24Ter	0.33
53	MPN	*ASXL1*	c.2077C>T	p.Arg693Ter	0.3
53	MPN	*MPL*	c.1543_1544delGTinsAA	W515K	0.71
54	MPN	*ASXL1*	c.1900_1922delAGAGAGGCGGCCACCACTGCCAT	p.Glu635ArgfsTer15	0.27
54	MPN	*MPL*	c.1543_1544delGTinsAA	W515K	91
55	MPN	*TET2*	c.2422delG	p.Glu808LysfsTer5	38.8
55	MPN	*CALR*	c.1099_1150delCTTAAGGAGGAGGAAGAAGACAAGAAACGCAAAGAGGAGGAGGAGGCAGAGG	p.Leu367ThrfsTer46	18.7
56	MPN	*JAK2*	c.1849G>T	V617F	0.45
57	MPN	*JAK2*	c.1849G>T	V617F	62.4
58	MPN	*JAK2*	c.1849G>T	V617F	0.62
59	MPN	*ASXL1*	c.1282C>T	p.Gln428Ter	41.9
59	MPN	*NRAS*	c.182A>G	Q61R	13.3
59	MPN	*SUZ12*	c.1956delA	p.Leu652PhefsTer9	40.1
60	MPN	*TET2*	c.4879C>T	p.Gln1627Ter	0.7
60	MPN	*CALR*	c.1099_1150delCTTAAGGAGGAGGAAGAAGACAAGAAACGCAAAGAGGAGGAGGAGGCAGAGG	p.Leu367ThrfsTer46	0.21
60	MPN	*UBE2T*	c.109+1G>A		0.49
61	MPN	*JAK2*	c.1849G>T	V617F	0.47
62	MPN	*TET2*	c.3101dupA	p.Phe1035ValfsTer8	0.41
62	MPN	*JAK2*	c.1849G>T	V617F	0.35
65	MPN	*MPL*	c.1543T>A	p.Trp515Arg	0.17
66	MPN	*PALB2*	c.1240C>T	p.Arg414Ter	40.4
66	MPN	*JAK2*	c.1849G>T	V617F	45.4
68	MPN	*JAK2*	c.1849G>T	V617F	32.9
70	AML	*CEBPA*	c.934_939delCAGAAG		12.5
70	AML	*MPL*	c.1069C>T	p.Arg357Ter	43.9
71	AML	*NPM1*	c.863_864insCAGA	p.Trp288CysfsTer12	13.2
71	AML	*TET2*	c.778_779delTT	p.Leu260ValfsTer3	29.7
72	AML	*ASXL1*	c.1934dupG	p.Gly646TrpfsTer12	35.5
72	AML	*XRCC2*	c.398_399delTA	p.Leu133ArgfsTer6	45.1
73	AML	*KIT*	c.2446G>T	D816Y	27
75	AML	*ATR*	c.5900delG	p.Gly1967ValfsTer7	53.1
75	AML	*DDX41*	c.1496dupC	p.Ala500CysfsTer9	47.7
77	AML	*NRAS*	c.35G>A	p.Gly12Asp	42.7
77	AML	*NPM1*	c.860_863dupTCTG	p.Trp288CysfsTer12	31.5
77	AML	*DNMT3A*	c.2207G>A	p.Arg736His	43.3
78	AML	*ASXL1*	c.2356dupA	p.Arg786LysfsTer3	42.2
78	AML	*IDH1*	c.394C>T	p.Arg132Cys	35.1
78	AML	*FAT1*	c.10993C>T	p.Arg3665Ter	41.9
69	MPN	*SBDS*	c.258+2T>C		45.9
79	MDS	*NPM1*	c.860_863dupTCTG	p.Trp288CysfsTer12	27.2
79	MDS	*BCOR*	c.4640-2A>G		65.3
80	AML	*PTPN11*	c.181G>T	p.Asp61Tyr	9.2

81	AML	*GATA1*	c.137_164delCCTCCACTGCCCCGAGCACAGCCACCGC	p.Ser46LeufsTer82	14.7
81	AML	*BRCA2*	c.8912delA	p.Lys2971SerfsTer5	49.9
81	AML	*EGLN1*	c.2T>G	p.Met1?	4.9
83	AML	*WT1*	c.1330A>T	p.Arg444Ter	3.6
85	AML	*GATA2*	c.1144-2A>G		41.2
85	AML	*NPM1*	c.863_864insCATG	p.Trp288CysfsTer12	33
87	MPN	*JAK2*	c.1849G>T	p.Val617Phe	38.4
89	AML	*ASXL1*	c.1925dupG	p.Gly643ArgfsTer15	11.2
89	AML	*ETV6*	c.472_473delGT	p.Val158ProfsTer10	43.5
89	AML	*EZH2*	c.1075A>T	p.Arg359Ter	46.2
89	AML	*NRAS*	c.35G>A	p.Gly12Asp	33.5
90	AML	*ASXL1*	c.2555C>A	p.Ser852Ter	42.3
90	AML	*LUC7L2*	c.727C>T	p.Arg243Ter	44.2
90	AML	*STAG2*	c.418C>T	p.Gln140Ter	76.2
90	AML	*NRAS*	c.181C>A	p.Gln61Lys	36.4
91	AML	*FLT3*	c.1824_1825insGGAAACGTTGATTTCAGAGAATATGAATATGATCTCAAATGGGAGTTTCCAAGAGAA	p.Glu608_Asn609insGlyAsnValAspPheArgGluTyrGluTyrAspLeuLysTrpGluPheProArgGlu	14.9
91	AML	*ASXL1*	c.1934dupG	p.Gly646TrpfsTer12	35.5
91	AML	*IDH2*	c.419G>A	p.Arg140Gln	44.6
91	AML	*RUNX1*	c.517_520dupTTCA	p.Thr174IlefsTer40	45.3
91	AML	*STAG2*	c.3097C>T	p.Arg1033Ter	99.1
92	AML	*ASXL1*	c.1934dupG	p.Gly646TrpfsTer12	36.8
93	AML	*NPM1*	c.860_863dupTCTG	p.Trp288CysfsTer12	28.9
93	AML	*FLT3*	c.1781_1782insTCGACAGGTGACCGGCTCCTCAGATAATGAGTACTTCTACGTTGATTT	p.Gln580_Arg595dup	10.2
93	AML	*CEBPA*	c.749_750delGC	p.Gly250GlufsTer70	10.7
93	AML	*CEBPA*	c.932A>C	p.Gln311Pro	34.8
93	AML	*IDH2*	c.419G>A	p.Arg140Gln	47.2
94	PMN	*CALR*	c.1099_1150delCTTAAGGAGGAGGAAGAAGACAAGAAACGCAAAGAGGAGGAGGAGGCAGAGG	p.Leu367ThrfsTer46	15.1
95	AML	*FLT3*	FLT3-ITD		10.3
96	AML	*WT1*	c.1110dupT	p.Val371CysfsTer14	49.3
96	AML	*PTEN*	c.146dupA	p.Asn49LysfsTer3	41.9
96	AML	*ASXL1*	c.1900_1922delAGAGAGGCGGCCACCACTGCCAT	p.Glu635ArgfsTer15	11
97	AML	*NF1*	c.3708+1G>T		26.8
97	AML	*IDH2*	c.419G>A	p.Arg140Gln	26.6
98	AML	*BCOR*	c.4072-1G>A		36.5
98	AML	*BCOR*	c.4038_4039delAG	p.Glu1348IlefsTer26	48.6
98	AML	*RAD21*	c.1161+1G>A		40.4
98	AML	*KRAS*	c.38G>A	p.Gly13Asp	39
99	AML	*DCLRE1C*	c.1696_1699dupAACA	p.Ser567LysfsTer12	
99	AML	*DDX41*	c.1496dupC	p.Ala500CysfsTer9	
100	AML	*CEBPA*	c.196_199dupGCCT	p.Tyr67CysfsTer42	40
100	AML	*CEBPA*	c.866G>C	p.Arg289Pro	41.5
100	AML	*TET2*	c.945delC	p.Gln317ArgfsTer30	42.6
100	AML	*TET2*	c.3247C>T	p.Gln1083Ter	41.4
101	MPN	*JAK2*	c.1849G>T	p.Val617Phe	69.9

101	MPN	*NF1*	c.3456dupA	p.Leu1153ThrfsTer42	31.5
103	MPN	*JAK2*	c.1849G>T	p.Val617Phe	93
103	MPN	*TET2*	c.5618T>C	p.Ile1873Thr	42.5
104	MPN	*JAK2*	c.1849G>T	p.Val617Phe	17.8
104	MPN	*ZRSR2*	c.715_716delTT	p.Phe239LeufsTer2	89.1
106	AML	*CEBPA*	c.262C>T	p.Gln88Ter	17
106	AML	*CEBPA*	c.914A>C	p.Gln305Pro	16.1
106	AML	*NRAS*	c.35G>A	p.Gly12Asp	17.2
106	AML	*LAMB4*	c.193-2A>C		19.5
107	AML	*NPM1*	c.860_863dupTCTG	p.Trp288CysfsTer12	28.5
107	AML	*TET2*	c.2964_2974delinsA	p.Pro989Valfs*15	22.2
108	AML	*IDH2*	c.419G>A	p.Arg140Gln	41.8
110	MPN	*JAK2*	c.1849G>T	p.Val617Phe	
111	MPN	*JAK2*	c.1849G>T	p.Val617Phe	31.9
111	MPN	*BRCA2*	c.10150C>T	p.Arg3384Ter	50.2
112	MPN	*JAK2*	c.1849G>T	p.Val617Phe	91.5
112	MPN	*IDH2*	c.419G>A	p.Arg140Gln	30.1
113	MPN	*JAK2*	c.1849G>T	p.Val617Phe	69.4
113	MPN	*ATRX*	c.3646A>G	p.Ile1216Val	100
113	MPN	*ADA*	c.659C>T	p.Ser220Leu	45
113	MPN	*BRCA2*	c.8954-5_8954-2delAACA		44.9
113	MPN	*DNMT1*	c.81-3delC		46.3
114	AML	*TP53*	c.583delA	p.Ile195SerfsTer52	53.8
114	AML	*BLM*	c.715G>A	p.Asp239Asn	44.9
114	AML	*GNAS*	c.1207_1208insCGACTCCGGGGCGGCCCGTGACGCCCCAGCCGATCC	p.Ile402_Gln403insProThrProGlyArgProValThrProGlnProIle	23.3
114	AML	*VPS13B*	c.1457T>G	p.Leu486Trp	54
114	AML	*STAG2*	c.1388C>T	p.Thr463Ile	49
114	AML	*NCOR2*	c.6626C>T	p.Thr2209Met	42.3
114	AML	*FLT3*	c.1333_1334delinsTT	p.Ala445Leu	38
114	AML	*UNC13D*	c.2136C>G	p.Ile712Met	52.3
115	AML	*FLT3*	c.1740_1793dupGGTGACCGGCTCCTCAGATAATGAGTACTTCTACGTTGATTTCAGAGAATATGA	p.Val581_Glu598dup	37.4
115	AML	*CEBPA*	c.713C>A	p.Ala238Glu	55.6
115	AML	*CDKN2A*	c.38C>A	p.Ala13Asp	42.7
115	AML	*NF1*	c.7540A>G	p.Thr2514Ala	47.9
115	AML	*SYNE1*	c.11067T>G	p.Ile3689Met	46.2
115	AML	*BCORL1*	c.4171G>A	p.Gly1391Arg	48.6
115	AML	*CREBBP*	c.5271C>T	p.Gly1757=	43.4
115	AML	*RNF168*	c.792A>G	p.Pro264=	44.8
116	AML	*CEBPA*	c.103_104insT	p.Arg35LeufsTer73	16.6
116	AML	*IDH2*	c.419G>A	p.Arg140Gln	17.4
116	AML	*BLM*	c.1544dupA	p.Asn515LysfsTer2	5.5
116	AML	*GATA2*	c.961C>T	p.Leu321Phe	18.2
116	AML	*NCOR2*	c.4576G>A	p.Ala1526Thr	61.9
116	AML	*KMT2C*	c.3401G>A	p.Ser1134Asn	6.1
116	AML	*SBF2*	c.1612G>T	p.Asp538Tyr	50.8
116	AML	*PCDHB1*	c.1591G>C	p.Val531Leu	54
116	AML	*SYNE1*	c.19348G>C	p.Glu6450Gln	53.2
117	AML	*XIAP*	c.697C>T	p.Arg233Trp	10.6
117	AML	*GPRC5A*	c.865G>T	p.Val289Leu	45.2

117	AML	*BLM*	c.2839A>G	p.Ile947Val	52.1
117	AML	*FANCL*	c.335C>T	p.Ser112Leu	46
118	AML	*TP53*	whole gene deletion (homozygous)		
118	AML	*SUZ12*	whole gene deletion		
118	AML	*NF1*	c.7330dupA	p.Thr2444AsnfsTer4	22.4
118	AML	*FANCG*	c.637_643delTACCGCC	p.Tyr213LysfsTer6	16.7
118	AML	*FAT1*	c.5671C>A	p.Pro1891Thr	45.8
118	AML	*EPOR*	c.1139C>T	p.Pro380Leu	48.4
118	AML	*NOTCH1*	c.5015G>A	p.Arg1672His	60
118	AML	*SLC37A4*	c.607G>A	p.Ala203Thr	47.1
118	AML	*FAT1*	c.1244A>G	p.Tyr415Cys	49.9
118	AML	*STX11*	c.668A>C	p.Asp223Ala	44
119	AML	*CEBPA*	c.68dupC	p.His24AlafsTer84	33
119	AML	*CEBPA*	c.907_909dupGCC	p.Ala303dup	39.1
119	AML	*DNMT1*	c.382C>G	p.Pro128Ala	51.5
119	AML	*SYNE1*	c.18467G>T	p.Arg6156Leu	45.9
119	AML	*GATA2*	c.961C>T	p.Leu321Phe	40.8
119	AML	*STXBP2*	c.1514C>T	p.Thr505Met	46.7
119	AML	*BRINP3*	c.1207C>G	p.Arg403Gly	43.8
119	AML	*FANCA*	c.953G>T	p.Arg318Met	47.4
119	AML	*FANCA*	c.527C>T	p.Ser176Phe	50
120	AML	*NPM1*	c.863_864insCATG	p.Trp288CysfsTer12	19.6
120	AML	*NRAS*	c.182A>G	p.Gln61Arg	37.8
120	AML	*TET2*	c.4877C>A	p.Thr1626Asn	45
120	AML	*NOTCH1*	c.4898G>A	p.Arg1633His	51.9
120	AML	*NTRK3*	c.1028G>A	p.Arg343Gln	48.8
120	AML	*JAK3*	c.637A>T	p.Ile213Phe	53.5
120	AML	*SYNE1*	c.3998G>A	p.Arg1333His	45.8
120	AML	*ABL1*	c.199G>A	p.Val67Ile	47.2
121	AML	*NPM1*	c.860_863dupTCTG	p.Trp288CysfsTer12	30.4
121	AML	*PTPN11*	c.1471C>A	p.Pro491Thr	35.3
121	AML	*IDH2*	c.419G>A	p.Arg140Gln	42.6
121	AML	*FLT3*	c.1724A>T	p.Gln575Leu	25.9
122	AML	*BCOR*	c.3646delA	p.Thr1216LeufsTer44	37.1
122	AML	*PTPN11*	c.182A>G	p.Asp61Gly	23.8
122	AML	*ETV6*	c.1141G>A	p.Gly381Arg	31
122	AML	*EGFR*	c.716G>C	p.Gly239Ala	50.9
122	AML	*XRCC2*	c.772C>T	p.Arg258Cys	50.4
123	AML	*EGFR*	c.2270A>G	p.Lys757Arg	46.2
123	AML	*FAT1*	c.7591A>G	p.Ile2531Val	52.2
123	AML	*KMT2C*	c.1181G>A	p.Cys394Tyr	6.7
123	AML	*CREBBP*	c.743C>T	p.Pro248Leu	44.9
124	AML	*CEBPA*	c.282_312delCGTGGGCCCCACGG GCGGCGGCGGCGGCGGC	p.Val95ThrfsTer55	22.7
124	AML	*CEBPA*	c.928_930dupACG	p.Thr310dup	33
124	AML	*WT1*	c.1144dupG	p.Ala382GlyfsTer3	39.9
124	AML	*WT1*	c.1289G>C	p.Arg430Pro	39.9
124	AML	*IKZF1*	c.14_17dupAGGG	p.Gln7GlyfsTer21	24.4
124	AML	*CDAN1*	c.1763T>C	p.Met588Thr	46.6
124	AML	*MYD88*	c.389T>C	p.Ile130Thr	46.7
125	AML	*TP53*	c.536A>G	p.His179Arg	34.9
125	AML	*TP53*	c.641A>G	p.His214Arg	35.3

125	AML	*SYNE1*	c.17644G>A	p.Glu5882Lys	34.5
125	AML	*LAMB4*	c.4438C>T	p.Leu1480Phe	73.7
126	MPN	*JAK2*	c.1849G>T	p.Val617Phe	54
126	MPN	*TERT*	c.838G>A	p.Glu280Lys	47.8
126	MPN	*RAD51*	c.1dupA	p.Met1?	49.2
126	MPN	*KMT2C*	c.2722G>T	p.Gly908Cys	8.1
126	MPN	*KMT2A*	c.6346A>C	p.Thr2116Pro	49.5
126	MPN	*GPRC5A*	c.371G>A	p.Arg124Gln	53.6
126	MPN	*IFNG*	c.53C>A	p.Ser18Tyr	39.2
126	MPN	*UNC13D*	c.602A>G	p.His201Arg	41.8
127	MPN	*CALR*	c.1103_1136delAGGAGGAGGAAGAAGACAAGAAACGCAAAGAGGA	p.Lys368ArgfsTer51	13.5
127	MPN	*KDR*	c.2668A>G	p.Ile890Val	44.8
127	MPN	*ETV6*	c.572G>T	p.Arg191Leu	53
127	MPN	*FANCI*	c.3907G>A	p.Glu1303Lys	48.6
127	MPN	*ERCC4*	c.1001C>T	p.Ser334Leu	43.2
127	MPN	*RTEL1*	c.604G>A	p.Val202Ile	36.1
127	MPN	*FAT1*	c.11818A>G	p.Thr3940Ala	50.3
128	AML	*FLT3*	c.1796_1837dupATGATCTCAAATGGGAGTTTCCAAGAGAAAATTTAGAGTTTG	p.Phe612_Gly613insAspAspLeuLysTrpGluPheProArgGluAsnLeuGluPhe	20.6
128	AML	*NPM1*	c.860_863dupTCTG	p.Trp288CysfsTer12	35.2
128	AML	*IDH1*	c.394C>G	p.Arg132Gly	46.9
128	AML	*CEBPA*	c.430dupG	p.Glu144GlyfsTer26	45.1
128	AML	*RAD21*	c.394C>T	p.Gln132Ter	46.6
128	AML	*ELANE*	c.407C>T	p.Ala136Val	39.8
128	AML	*ATRX*	c.2234G>A	p.Ser745Asn	100
128	AML	*MRE11A*	c.1199G>C	p.Arg400Thr	48.7
128	AML	*FAT1*	c.755A>C	p.Glu252Ala	44.9
129	MPN	*JAK2*	c.1849G>T	p.Val617Phe	18.8
129	MPN	*VPS13B*	c.7333G>A	p.Glu2445Lys	51.2
129	MPN	*P2RY2*	c.907C>T	p.Pro303Ser	42.8
129	MPN	*NCOR2*	c.2420C>T	p.Thr807Met	27.6
129	MPN	*KMT2C*	c.2855A>G	p.Lys952Arg	50.4
130	AML	*FLT3*	c.1834_1835dupACTTCTACGTTGATTTCAGAGAATATGAATATGATCTCAAATGGGAGTTTCCAAGAGAAAATTTAGAGT		11.2
130	AML	*KMT2A*	exon 3-6 duplication		
130	AML	*GNAS*	c.245A>G	p.Asn82Ser	50.3
130	AML	*NCOR2*	c.3986-8C>T		47.2
130	AML	*NOTCH1*	c.6941T>A	p.Leu2314Gln	45.6
130	AML	*NCOR2*	c.2890A>C	p.Lys964Gln	44.8
130	AML	*FANCI*	c.3907G>A	p.Glu1303Lys	49.4
131	AML	*WT1*	c.1018G>T	p.Glu340Ter	19.2
131	AML	*OR8B12*	c.584A>G	p.Glu195Gly	49.9
131	AML	*SYNE1*	c.4900C>G	p.Gln1634Glu	32.5
131	AML	*ABCB7*	c.667A>G	p.Ile223Val	51
132	AML	*NRAS*	c.35G>A	p.Gly12Asp	28.9
132	AML	*EZH2*	c.45G>A	p.Trp15Ter	6.4
132	AML	*TP53*	c.818G>A	p.Arg273His	37.8
132	AML	*DNMT3A*	c.2204A>G	p.Tyr735Cys	39.7

132	AML	*SETBP1*	c.2620G>A	p.Asp874Asn	38.1
132	AML	*U2AF1*	c.470A>C	p.Gln157Pro	43.6
132	AML	*ATRX*	c.4025G>A	p.Arg1342Gln	98.8
132	AML	*ETNK1*	c.731A>G	p.Asn244Ser	39.3
132	AML	*KLF1*	c.790T>C	p.Ser264Pro	42.2
132	AML	*KMT2C*	c.5512C>T	p.Pro1838Ser	20.6
133	AML	*KMT2A*	exon 2-8 duplication		
133	AML	*DNMT3A*	c.1936+2_1936+14delTGAGGGGTG CAGG		10.8
133	AML	*IDH2*	c.515G>A	p.Arg172Lys	31.5
133	AML	*ATR*	c.7370A>G	p.Tyr2457Cys	27.2
133	AML	*PRPF40B*	c.1893G>C	p.Glu631Asp	48.1
134	AML	*KRAS*	c.35G>A	p.Gly12Asp	12.2
134	AML	*BRCA2*	c.7052C>G	p.Ala2351Gly	48.4
134	AML	*DNMT1*	c.470C>T	p.Ala157Val	56.1
134	AML	*RTEL1*	c.3299G>A	p.Cys1100Tyr	49.6
134	AML	*LAMB4*	c.4754G>A	p.Arg1585Gln	48.4
135	AML	*NRAS*	c.182A>G	p.Gln61Arg	9.9
135	AML	*ASXL1*	c.2558C>T	p.Pro853Leu	19.7
135	AML	*U2AF1*	c.101C>A	p.Ser34Tyr	37.6
135	AML	*ANKRD26*	c.391A>G	p.Ile131Val	43.3
135	AML	*LAMB4*	c.911C>T	p.Pro304Leu	47.6
136	AML	*ETV6*	c.1062C>G	p.Tyr354Ter	25
136	AML	*BRCA2*	c.10150C>T	p.Arg3384Ter	52.5
136	AML	*NF1*	c.1885G>A	p.Gly629Arg	17.1
136	AML	*ETV6*	c.1195C>T	p.Arg399Cys	12.1
136	AML	*BRCA1*	c.2286A>T	p.Arg762Ser	47.7
136	AML	*KMT2C*	c.5717G>A	p.Arg1906Gln	44.6
137	MPN	*CALR*	c.1099_1150delCTTAAGGAGGAGG AAGAAGACAAGAAACGCAAAG AGGAGGAGGAGGCAGAGG	p.Leu367ThrfsTer46	11.3
137	MPN	*RAD50*	c.3836G>A	p.Arg1279His	46.3
138	MDS	*BCOR*	c.2488_2489delAG	p.Ser830CysfsTer6	86.6
138	MDS	*PTPN11*	c.218C>T	p.Thr73Ile	9.2
138	MDS	*PHF6*	c.241-8_244delTGCTGTAGATGT		76.3
138	MDS	*NRAS*	c.35G>C	p.Gly12Ala	8.4
138	MDS	*NRAS*	c.37G>C	p.Gly13Arg	17.7
138	MDS	*RUNX1*	c.496C>T	p.Arg166Ter	49.4
138	MDS	*EZH2*	c.1876G>A	p.Val626Met	75.5
138	MDS	*SMC3*	c.2338G>A	p.Glu780Lys	24.3
138	MDS	*EZH2*	c.838T>C	p.Ser280Pro	5.4
138	MDS	*ETV6*	c.1196G>A	p.Arg399His	40.2
138	MDS	*IDH2*	c.568G>A	p.Gly190Ser	11.3
138	MDS	*RTEL1*	c.1478G>A	p.Arg493His	42.7
138	MDS	*ETNK1*	c.731A>G	p.Asn244Ser	38.5
138	MDS	*CDAN1*	c.2066C>T	p.Ala689Val	50
139	MPN	*JAK2*	c.1849G>T	p.Val617Phe	35.7
139	MPN	*PDGFRA*	c.1757G>A	p.Trp586Ter	33
139	MPN	*XIAP*	c.1100A>G	p.Asp367Gly	46.9
