# Supplementary material for: Targeted next generation sequencing can serve as an alternative to conventional tests in myeloid neoplasms
Source: PLoS One. 2019 Mar 6;14(3):e0212228. doi: 10.1371/journal.pone.0212228 (PMC6402635; doi:10.1371/journal.pone.0212228)
Supplement: S5 Table — (PDF) [file pone.0212228.s005.pdf]

S5 Table. Concordance rates between Sanger sequencing and NGS results

***NPM1* exon 12**		**NGS**	
		**Positive**	**Negative**
**Sanger**	**Positive**	5	-
	**Negative**	-	20

CEBPA		**NGS**	
		**Positive**	**Negative**
**Sanger**	**Positive**	3	-
	**Negative**	-	21

***IDH1* exon 4**		**NGS**	
		**Positive**	**Negative**
**Sanger**	**Positive**	2	-
	**Negative**	-	15

***IDH2* exon 4**		**NGS**	
		**Positive**	**Negative**
**Sanger**	**Positive**	3	-
	**Negative**	-	14

***DNMT3A* exon 23**		**NGS**	
		**Positive**	**Negative**
**Sanger**	**Positive**	3	-
	**Negative**	-	14

***ASXL1* exon 12**		**NGS**	
		**Positive**	**Negative**
**Sanger**	**Positive**	3	-
	**Negative**	-	14

***FLT3* ITD**

		**NGS**	
		**Positive**	**Negative**
**Sanger**	**Positive**	8*	-
	**Negative**	-	18

* By Pindel algorithm
