# Supplementary material for: Targeted next generation sequencing can serve as an alternative to conventional tests in myeloid neoplasms
Source: PLoS One. 2019 Mar 6;14(3):e0212228. doi: 10.1371/journal.pone.0212228 (PMC6402635; doi:10.1371/journal.pone.0212228)
Supplement: S6 Table — (PDF) [file pone.0212228.s006.pdf]

S6 Table. The results of chromosomal CNVs and conventional karyotyping

Case	Disease	NGS, CNV analysis with CopywriteR	Conventional karyotyping	FISH
P38	AML	del 5q14.3q33.3, 7p12.2q36.3 17p	45,XX,del(3)(p14),inv(3)(q21q26),der(5)t(3;5)(p21;q22),der(7;17)(p10;q10)[20]	No abnormalities
P47	AML	9q 21.2q31.1 interstitial deletion	45,X,-Y,t(8;21)(q22;q22)[21]	t(8:21)
P19	AML	9p21-ter duplication, 3q26.3 deletion	Not tested	Inversion or translocation involving CBFB at 16q22
P14	AML	8q13-ter duplication	47,XX,+8,t(15;17)(q24;q21)[20]	Trisomy 8 or Tetrasomy 8. Translocation involving t(15;17).
P35	AML	7q31.33-ter deletion	46,XX,del(7)(q31)[6]/46,XX[14]	del7q
P2	AML	7q22-ter deletion, 3p13 interstitial deletion	46,XX,del(7)(q22)[9]/46,XX[19]	No abnormalities
P76	AML	-7q22	46,XX,del(7)(q22)[6]/46,XX[28]	No abnormalities
P82	AML	-7q	46,XY,t(15;17)(q24;q21)[5]/46,idem,del(7)(q22)[7]/46,XY[1]	t(15:17)
P83	AML	-7p,-7q	46,XY,t(15;17)(q24;q21)[16]/46,XY[4]	t(15:17)
P52	MDS	5q23.2q33.2 deletion, -7, 17p+, 20q11.22-ter deletion, 22q+	45-47,XX,del(5)(q31),-7,-20,+22,+2mar[cp18]/46,XX[2]	Not tested
P48	AML	5q-, 7q-, 9p21.1q32 deletion, 16q12.2-ter deletion,19q12q13.2 deletion, 22q13.31-ter deletion	41~44,XY,+del(3)(q13),del(5),del(7)(q22),-9,add(11),-13,add(18)[cp29]	-9 and -16
P42	AML	5p+ 9p+, 5q-, 7q-, 9q-	complex karyotype including del(5q), del(7), del(9q), and -21	+21 and -9
P20	AML	1q21.3-ter duplication, 16q deletion	46,XX,+del(1)(p13),-16,der(21)t(16;21)(p11;q22)	Monosomy 16
P49	AML	1q+, 4q-, 5p-, 10q-, 16q+, 17q-	complex karyotype	+16 and -17
P81	AML	1q+, 18q22q23 deletion, +21	46,XY,der(13;21)(q10;q10)c,der(18)t(1;18)(q12;q22),+21c[24]	+21
P9	AML	13q14-21 deletion, 5q31.2 deletion	46,XY[13]	Translocation involving 9q34
P90	AML	12p12.3p13.2 interstital deletion	46,XY,del(12)(p11p13)[20]	Not tested
P80	AML	+8,+11q23.3-ter, +19	48,XY,+8,t(11;19)(q23;p13),+der(19)t(11;19)[24]	KMT2A Break apart positive
P26	AML	+8, 7q36.1 deletion	47,XX,+8,inv(16)(p13q22)[22]	inv(16), +8
P97	AML	+8, 5q21.2-ter deletion,7q22-ter deletion	Complex karyotype	Not tested
P15	AML	+8	46,XY,t(11;17)(q23;q21)[3]/47,sl,+8[7]/46,XY[10]	Translocation involving RARA at 17q21
P51	MDS	+8	47,XY,+8[20]	+8
P89	AML	+8	47,XY,+8[14]/46,XY[7]	+8

P106	AML	+4, +10q, -18q	47,XY,+4,der(18)t(10;18)(q21;q21.1)[3]/46,XY[17]	Not tested
P107	AML	+4	47,XY,+4[13]	Not tested
P44	AML	+21	46,XX,13pstk+,+21,der(21;21)(q10;q10)[3]/46,XX,13pstk+[6]	+21
P85	AML	+13	47,XY,+13[17]/ 46,XX[3]	No abnormalities
P102	AML	1q+, +8, 6q12q16 deletion, 7q-	47,XY,+1,der(1;7)(q10;p10),del(6)(q23),+8[20]	+8
P11	AML	Y loss	45,X,-Y,t(8;21)(q22;q22)[20]/46,XY[4]	Translocation involving t(8;21)
P23	AML	-Y	45,X,-Y,t(8;21)(q22;q22)[20]	Translocation involving t(8;21)
P37	AML	Normal	47,XX,+21[11]/46,XX[19]	+21
P40	AML	Normal	47,XY,der(1)t(1;1)(p36.3;q24),+8[37]/46,XY[5]	No abnormalities
P41	AML	Normal	47,XY,+13[11]/47,XY,+15[4]/46,XY[14]	+15
P43	AML	Normal	46,XY,del(9)(q22)[6]/46,XY[17]	Not tested
P92	AML	Normal	47,XX,+11[8]/46,XX[11]	+11
P12	AML	Normal	46,XY,t(15;17)(q24;q21)[7]	Translocation involving t(15;17)
P13	AML	Normal	46,XX,t(15;17)(q24;q21)[19]/46,XX[1]	Translocation involving t(15;17)
P72	AML	Normal	46,XX,t(8;21)(q22;q22)[20]	t(8:21)
P73	AML	Normal	46,XX,t(8;21)(q22;q22)[20]	t(8:21)
P84	AML	Normal	46,XY,t(15;17)(q24;q21)[16]/46,XY[4]	t(15:17)
P36	AML	Normal	46,XX,inv(16)(p13q22)[19]/46,XX[9]	inv(16),t(16;16) 60.3%
P45	AML	Normal	46,XX,t(15;17)(q24;q21)[22]/46,XX[2]	t(15;17)
P1	AML	Normal	46,XY[20]	No abnormalities
P5	AML	Normal	46,XY[21]	No abnormalities
P6	AML	Normal	46,XY[21]	No abnormalities
P7	AML	Normal	46,XX[11]	No abnormalities
P3	AML	Normal	46,XY[20]	No abnormalities
P4	AML	Normal	46,XX[21]	No abnormalities
P28	AML	Normal	46,XY[7]	No abnormalities
P10	AML	Normal	46,XY,1qh+[17]	No abnormalities
P54	MPN	Normal	46,XX[20]	Not tested
P55	MPN	Normal	46,XY[14]	Not tested

P56	MPN	Normal	46,XX[20]	Not tested
P57	MPN	Normal	46,XY[20]	Not tested
P93	AML	Normal	46,XY[20]	Not tested
P94	MPN	Normal	46,XY[20]	Not tested
P95	AML	Normal	46,XY[20]	Not tested
P96	CMML	Normal	46,XY[20]	Not tested
P98	AML	Normal	46,XY[20]	No abnormalities
P99	AML	Normal	46,XY[20]	No abnormalities
P100	AML	Normal	46,XY[20]	-8
P101	MPN	Normal	46,XY[20]	Not tested
P58	MPN	Normal	46,XX[20]	Not tested
P59	aCML	Normal	46,XY[20]	Not tested
P60	MPN	Normal	46,XX[17]	Not tested
P61	MPN	Normal	46,XX[20]	Not tested
P62	MPN	Normal	46,XX[20]	Not tested
P63	MPN	Normal	46,XX[20]	Not tested
P64	MPN	Normal	46,XX[20]	Not tested
P65	MPN	Normal	46,XX[20]	Not tested
P30	MDS	Normal	46,XY[20]	No abnormalities
P31	AML	Normal	46,XX[24]	No abnormalities
P32	AML	Normal	46,XX[24]	No abnormalities
P24	AML	Normal	46,XY[20]	No abnormalities
P25	AML	Normal	46,XY[23]	No abnormalities
P17	AML	Normal	46,XX[20]	Amplification involving RUNX1T1 at 8q21.3 or trisomy 8
P69	MPN	Normal	46,XY[20]	Not tested
P77	AML	Normal	46,XY[20]	No abnormalities
P78	AML	Normal	46,XY[20]	No abnormalities
P79	MDS	Normal	46,XY[20]	No abnormalities
P103	MPN	Normal	46,XY[20]	Not tested

P104	MPN	Normal	46,XY[20]	Not tested
P105	MPN	Normal	46,XY[20]	Not tested
P109	MPN	Normal	46,XY[20]	Not tested
P110	MPN	Normal	46,XY[20]	Not tested
P111	MPN	Normal	46,XX[20]	Not tested
P87	mpn	Normal	46,XY[20]	Not tested
P112	MPN	Normal	46,XY[20]	Not tested
P68	MPN	Normal	46,XY[20]	Not tested
P71	AML	Normal	46,XX[22]	Not tested
P75	AML	Normal	46,XY[36]	No abnormalities
P39	AML	Normal	46,XY[22]	Not tested
P70	AML	Normal	46,XX[23]	No abnormalities
P34	AML	Normal	46,XY[20]	Translocation involving t(8;21)
P66	MPN	Normal	46,XY[20]	Not tested
P22	AML	Normal	46,XY[20]	No abnormalities
P18	AML	Normal	46,XX[20]	Translocation involving MLL at 11q23
P53	MPN	Normal	ND	Not tested
P8	AML	Normal	Not interpretable result	No abnormalities
P16	AML	Normal	Not interpretable result	No abnormalities
P91	AML	Normal	Not interpretable result	No abnormalities
P27	AML	Normal	Not interpretable result	Triple translocation involving t(8;21;v) is suspected.
